# Endothelial VEGFR2-PLC**γ** signaling regulates vascular permeability and antitumor immunity through eNOS/Src

**DOI:** 10.1172/JCI161366

**Published:** 2023-10-16

**Authors:** Elin Sjöberg, Marit Melssen, Mark Richards, Yindi Ding, Catarina Chanoca, Dongying Chen, Emmanuel Nwadozi, Sagnik Pal, Dominic T. Love, Takeshi Ninchoji, Masabumi Shibuya, Michael Simons, Anna Dimberg, Lena Claesson-Welsh

**Affiliations:** 1 Department of Immunology, Genetics and Pathology, Beijer and Science for Life Laboratories, Uppsala University, Uppsala, Sweden.; 2 Yale Cardiovascular Research Center, Department of Internal Medicine, Yale University School of Medicine, New Haven, Connecticut, USA.; 3 Institute of Physiology and Medicine, Jobu University, Takasaki, Gunma, Japan.; 4 Department of Cell Biology, Yale University School of Medicine, New Haven, Connecticut, USA.

**Keywords:** Oncology, Vascular Biology, Signal transduction

## Abstract

Endothelial phospholipase Cγ (PLCγ) is essential for vascular development; however, its role in healthy, mature, or pathological vessels is unexplored. Here, we show that PLCγ was prominently expressed in vessels of several human cancer forms, notably in renal cell carcinoma (RCC). High PLCγ expression in clear cell RCC correlated with angiogenic activity and poor prognosis, while low expression correlated with immune cell activation. PLCγ was induced downstream of vascular endothelial growth factor receptor 2 (VEGFR2) phosphosite Y1173 (pY1173). Heterozygous *Vegfr2^Y1173F/+^* mice or mice lacking endothelial PLCγ (*Plcg1^iECKO^*) exhibited a stabilized endothelial barrier and diminished vascular leakage. Barrier stabilization was accompanied by decreased expression of immunosuppressive cytokines, reduced infiltration of B cells, helper T cells and regulatory T cells, and improved response to chemo- and immunotherapy. Mechanistically, pY1173/PLCγ signaling induced Ca^2+^/protein kinase C–dependent activation of endothelial nitric oxide synthase (eNOS), required for tyrosine nitration and activation of Src. Src-induced phosphorylation of VE-cadherin at Y685 was accompanied by disintegration of endothelial junctions. This pY1173/PLCγ/eNOS/Src pathway was detected in both healthy and tumor vessels in *Vegfr2^Y1173F/+^* mice, which displayed decreased activation of PLCγ and eNOS and suppressed vascular leakage. Thus, we believe that we have identified a clinically relevant endothelial PLCγ pathway downstream of VEGFR2 pY1173, which destabilizes the endothelial barrier and results in loss of antitumor immunity.

## Graphical abstract

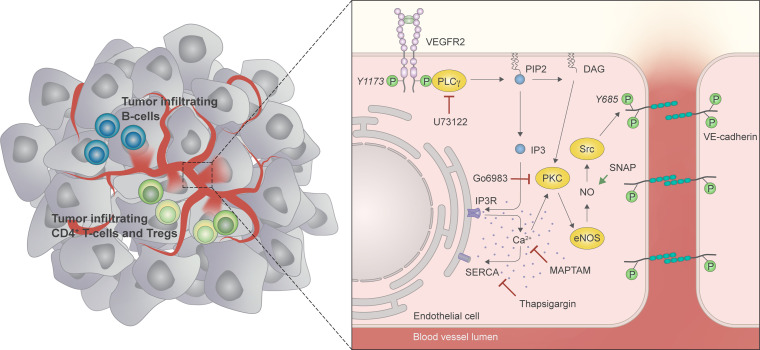

## Introduction

Growth of solid cancers is accompanied by hypoxia-driven formation of abnormal blood vessels with a defective endothelial barrier, often due to chronic exposure to endothelial cell ligands such as vascular endothelial growth factor A (VEGFA) (1, 2). Barrier deterioration is manifested as gaps between endothelial cells (ECs) and enhanced exchange of molecular mediators and cells across the vessel wall, resulting in tumor edema, attenuated drug delivery, suppressed antitumoral immune response, and increased metastatic spread (1, 3). The EC barrier is normally maintained via cell-cell junctions, including adherens junctions, which are composed of homophilic complexes of VE-cadherin. In response to VEGFA, VE-cadherin is phosphorylated and internalized, accompanied by dismantling of adherens junctions (4). By therapeutic targeting of VEGFA or its main receptor, VEGF receptor-2 (VEGFR2), the barrier can be stabilized, resulting in reduced interstitial pressure, increased perfusion of tumor vessels, decreased malignant invasion and metastatic spread, enhanced infiltration and activation of immune cells, and improved therapy response (5, 6).

However, it is also well established that drugs targeting VEGFA/VEGFR2 (anti-VEGFs), have deleterious effects on the vasculature in the long run, eventually resulting in vascular pruning and endothelial cell death (7). Moreover, animal models have demonstrated that efficient suppression of VEGFA/VEGFR2 signaling by tyrosine kinase inhibitors may promote disease progression by increasing hypoxia, leading to malignant invasion and increased metastatic spread (8–10). Blockade of individual signal transduction pathways and their corresponding endothelial response downstream of VEGFR2, such as vascular leakage, is an attractive, alternative therapeutic strategy.

Upon VEGF binding, VEGFR2 is activated and autophosphorylated on several tyrosine residues, including Y949 in the kinase insert, Y1052/Y1057 in the kinase domain, and Y1173 and Y1212 in the C-terminal tail — these are mouse sequence numbers; human sequence numbers are +2 residues, i.e. Y951, etcetera — initiating distinct downstream signaling pathways. The phosphosite Y949 (pY949) site binds T cell specific adaptor (TSAd), which mediates activation of Src family kinases (SFKs) (11). Mutation of *Vegfr2* Y949 to phenylalanine (F) is fully compatible with embryonic development but results in suppression of vascular permeability and decreased metastatic spread in cancer-challenged mice (12). The pY1052/Y1057 sites are required for induction of kinase activity (13). Signaling downstream of the pY1212 site is required for Myc activation and endothelial proliferation during development (14). Signaling downstream of the pY1173 site is necessary for endothelial progenitor differentiation; mice expressing a constitutive tyrosine-phenylalanine exchange mutation at *Vegfr2* Y1173 (Y1173F) die at E8.5–E9.5, due to arrested vascular development (15, 16). pY1173 recruits phospholipase Cγ (PLCγ), which regulates hydrolysis of phosphatidyl 2,3 bisphosphate in the plasma membrane. This results in generation of diacylglycerol (DAG) and downstream activation of protein kinase C (PKC), as well as inositol 1,4,5 trisphosphate, regulating Ca^2+^ release from the endoplasmic reticulum. Of these downstream pathways, PKC-mediated activation of extracellular regulated kinases 1/2 (ERK1/2) plays a critical role in VEGF-induced endothelial proliferation (17). Despite the well understood role of pY1173 signaling in embryonic development, information is lacking regarding signaling related to this phosphosite in the adult vasculature.

Here, we identify an essential role of pY1173/PLCγ/endothelial nitric oxide synthase–dependent (eNOS-dependent) signaling in the adult vasculature regulating endothelial barrier integrity. Activated eNOS, phosphorylated on S1176, induced Src-mediated tyrosine phosphorylation of VE-cadherin, accompanied by its internalization and disintegration of endothelial adherens junctions. This pathway operates in the healthy endothelium as well as in tumor vessels. Tumor vessels in human cancer express PLCγ, and, in clear cell renal cell carcinoma (ccRCC), PLCγ serves as an independent biomarker for poor survival. In mouse models, the Y1173F mutation suppresses tumor vessel leakage, accompanied by vessel normalization, reduced infiltration of immune cells, and reduced production of immunosuppressive cytokines. Thus, the identified VEGF-R2 Y1173/PLCγ/eNOS/Src signaling pathway regulates both normal and pathological vascular leakage. This information could be exploited for drug development with the aim to alleviate vascular leakage and immunosuppression in ccRCC.

## Results

### Clinical relevance of tumor endothelial PLCγ in kidney cancer.

**Figure 1:**
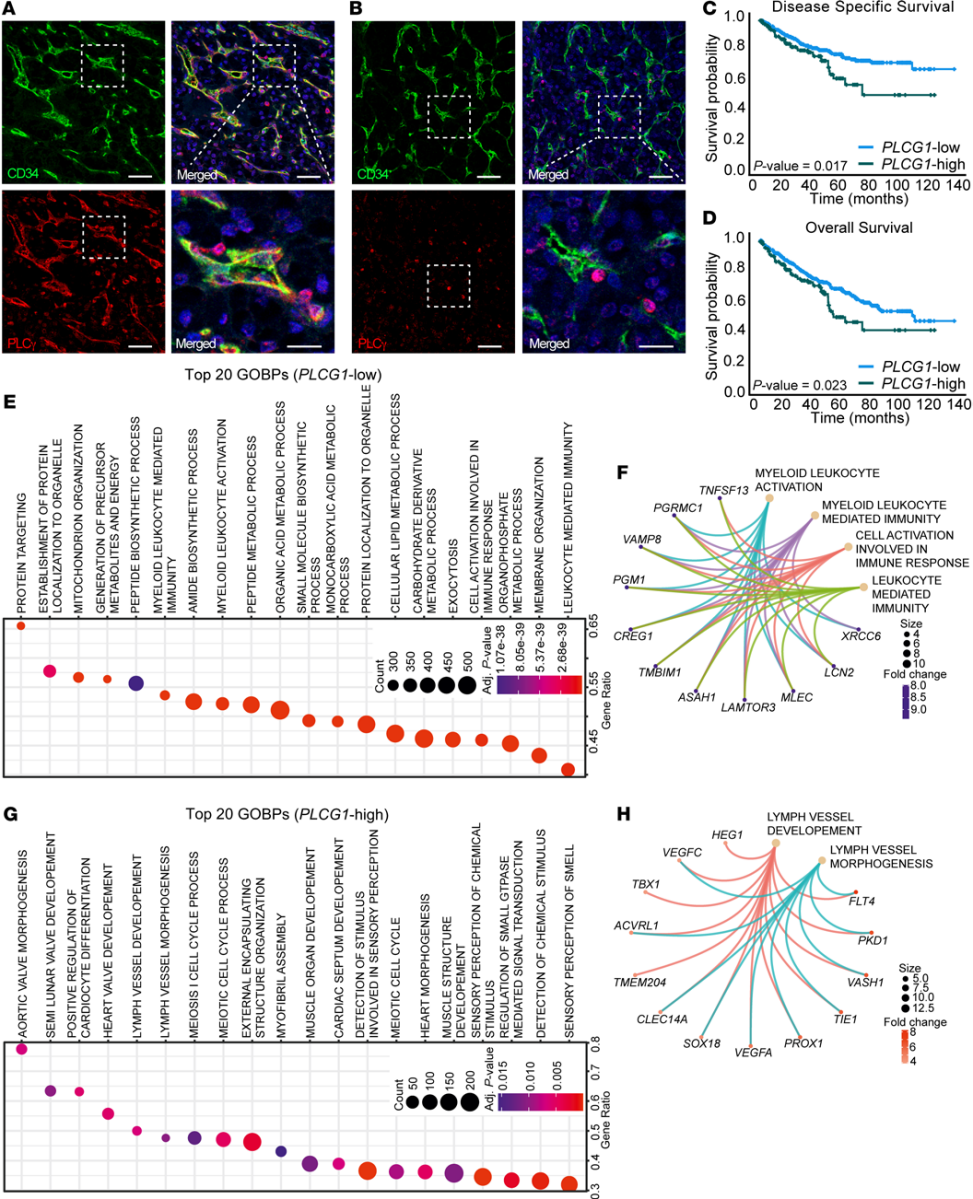
Tumor endothelial PLCγ expression correlates with worse patient survival and biological processes in patients with ccRCC. (**A** and **B**) Representative images of patients with RCC positive (*n* = 5, **A**) and negative (*n* = 7; **B**) for vascular PLCγ expression. Scale bar: 50 μm. Lower right panels show magnifications from insets. Scale bar, 20 μm. (**C** and **D**) Kaplan-Meier curves showing correlative analysis of *PLCG1* mRNA expression and disease specific survival (**C**) and overall survival (**D**) in the ccRCC TCGA data set (KIRC); *n* = 383 (*PLCG1*-low subgroup) and 127 patients (*PLCG1*-high subgroup). (**E**) Ranking of the 20 (GOBPs most enriched in the *PLCG1*-low subgroup (*n* = 383 patients) based on significance (*P_adj_* shown as heatmap) and ratio of affected genes within each GOBP. Overrepresentation analysis was performed based on a hypergeometric distribution corresponding to the 1-sided Fisher’s exact test (ClusterProfiler). (**F**) CNET plot showing networks of top differentially expressed genes (DEGs) in the immune activity GOBPs listed in **E**. (**G**) Ranking as in **E**, of the 20 most enriched GOBPs in the *PLCG1*-high subgroup (*n* = 127 patients). (**H**) CNET plot showing network of top DEGs in the indicated GOBPs in **G**.

The contribution of VEGFR2/PLCγ signaling in the adult endothelium, healthy or disease-afflicted, is unknown. As a basis for hypothesis-driven exploration of the role of endothelial PLCγ signaling, we investigated endothelial PLCγ expression in human cancer. PLCγ immunostaining of human tumor tissue microarrays (TMAs) — composed of RCC, skin cancer, melanoma, lung cancer, and pancreatic cancer — revealed endothelial expression of PLCγ to different extents, in addition to expression in tumor cells and immune cells in the different cancer forms (Supplemental Figure 1A; supplemental material available online with this article; https://doi.org/10.1172/JCI161366DS1). In 5 of 12 examined biopsies from patients with RCC, PLCγ was prominently expressed in the tumor endothelium, with minor expression in immune cells (Figure 1A and Supplemental Figure 1A). Interestingly, certain patients with RCC lacked vascular PLCγ, but the expression in immune cells remained (Figure 1B). Given the marked endothelial expression in a subgroup of patients, RCC was chosen for extended analysis.

Clinical features correlating with overall PLCγ expression were explored using the gene expression TCGA-data set of 510 cases of ccRCC (KIRC) (https://www.cancer.gov/ccg/research/genome-sequencing/tcga). The expression cut-off was determined by Kaplan Meier-analysis of patients divided into 4 equal subgroups (25% of patients in each subgroup) based on quartiles of *PLCG1* expression (subgroup 1: low expression; subgroup 2: mid/low expression; subgroup 3: mid/high expression; subgroup 4: high expression). Patients in subgroup 4 showed a particularly poor outcome compared with subgroups 1–3 which showed similar outcomes (Supplemental Figure 1B). Therefore, patients with expression levels above and below the third quartile were assigned as *PLCG1*-high (subgroup 4; 127 patients) and *PLCG1*-low expressing patients (subgroup 1–3; 383 patients), respectively. There was no association of the *PLCG1*-high subgroup with any clinicopathological characteristics analyzed (Supplemental Table 1). Survival analyses, however, revealed a significant correlation of high *PLCG1* mRNA levels and worse disease-specific survival (DSS) (Figure 1C) as well as overall survival (OS) (Figure 1D), with an increased risk of death (DSS: HR =1.645 *P* = 0.018; OS: HR = 1.481, *P* = 0.024). PLCγ expression was further identified as an independent marker for poor ccRCC outcome. The prognostic significance of the *PLCG1*-high subgroup remained in multivariable analyses adjusted for clinicopathological characteristics including sex, age, tumor-, nodal- and metastasis stage (DSS: HR = 1.642, *P* = 0.022; OS: HR = 1.545, *P* = 0.014) (Table 1). In summary, our analyses identified PLCγ as a clinically relevant biomarker for outcomes for patients with ccRCC.

### Biological processes in RCC correlating with PLCγ transcript levels.

Given the clinical relevance of PLCγ expression in RCC, the potential disease impact of differentially expressed genes (DEGs) with a log_2_ fold change of more than 1.0 or less than –1.0 in the *PLCG1*-high and -low subgroups in the KIRC cohort (Supplemental Data File 1) was assessed by examining enrichment of gene ontology biological processes (GOBPs). GOBPs were ranked based on significance (adjusted *P* value shown as heatmap) and ratio of affected genes within each GOBP. In the *PLCG1*-low group GOBPs were enriched for metabolic processes (12 of the 20 top GOBPs) and for immune activation (4 of the 20 top GOBPs) (Figure 1E). Of all enriched GOBPs in the *PLCG1*-low subgroup, 54 (5.2%) were related to immunity and inflammation (Supplemental Figure 1C). Lead DEGs in the immunity and inflammatory GOBPs enriched in the *PLCG1*-low subgroup were largely overlapping and included genes such as *TNFSF13* (*APRIL*), implicated in B cell survival and differentiation in RCC (18), as well as *ASAH1* (19) and *XRCC6* (20), implicated in T cell function (Figure 1F). In contrast, GOBPs enriched in the *PLCG1*-high subgroup related to proliferative and morphogenic processes, including lymph vessel morphogenesis, in agreement with the loss of the hypoxia-regulated von Hippel Lindau (vHL) tumor suppressor and hypoxia-induced expression of VEGFA in ccRCC (21) (Figure 1, G and H). Strikingly, there was no enrichment of immune activity–related GOBPs in the *PLCG1*-high subgroup, and angiogenic GOBPs were missing in the *PLCG1*-low subgroup.

It is increasingly appreciated that tumor vascular dysfunction hallmarks such as vessel fragility, hyperpermeability and poor perfusion steer tumor metabolism (22) as well as tumor immune cell infiltration and function, from antitumor to protumor directed effects (6). Thus, the GOBPs enriched in the *PLCG1-*low and -high RCC subgroups — metabolism and immune activation, and proliferation and morphogenesis, respectively — could all be related directly or indirectly to tumor vascular features.

### VEGFR2 Y1173 heterozygosity accompanied by decreased tumor vascular permeability and enhanced antitumor immunity.

PLCγ is a binding partner for the VEGFR2 phosphosite pY1173, which regulates endothelial cell differentiation (23), but no unbiased approach has been applied to identify the interactome of this phosphosite. To this end, biotinylated peptides corresponding to the region around Y1175 in the human sequence, corresponding to mouse Y1173, phosphorylated or not, were used to capture downstream signal transducers from human endothelial cell lysates. Subsequent mass spectrometry (MS) analysis identified PLCγ as the main interaction partner for pY1175 (Supplemental Figure 2A). RASA1, SHP2 (*PTPN11*), VAV2, and CSK also bound efficiently to the pY1175 peptide (Supplemental Figure 2A). Western blot analysis corroborated a predominant binding of PLCγ to the pY1175 peptide compared with the pY951 and pY1214 peptides (Supplemental Figure 2B). In agreement, mouse aortic endothelial (MAE) cells expressing mouse WT VEGFR2 showed potent accumulation of PLCγ pY783 (denoted pPLCγ below) required for enzyme activation, which was lacking in MAE cells expressing the VEGFR2 Y1173F mutant (Supplemental Figure 2, C–E). The suppressed PLCγ phosphorylation was not due to loss of other receptor phosphosites; VEGFR2 pY949 and pY1212 were still induced in MAE-Y1173F cells (Supplemental Figure 2, C, F, and G).

**Figure 2:**
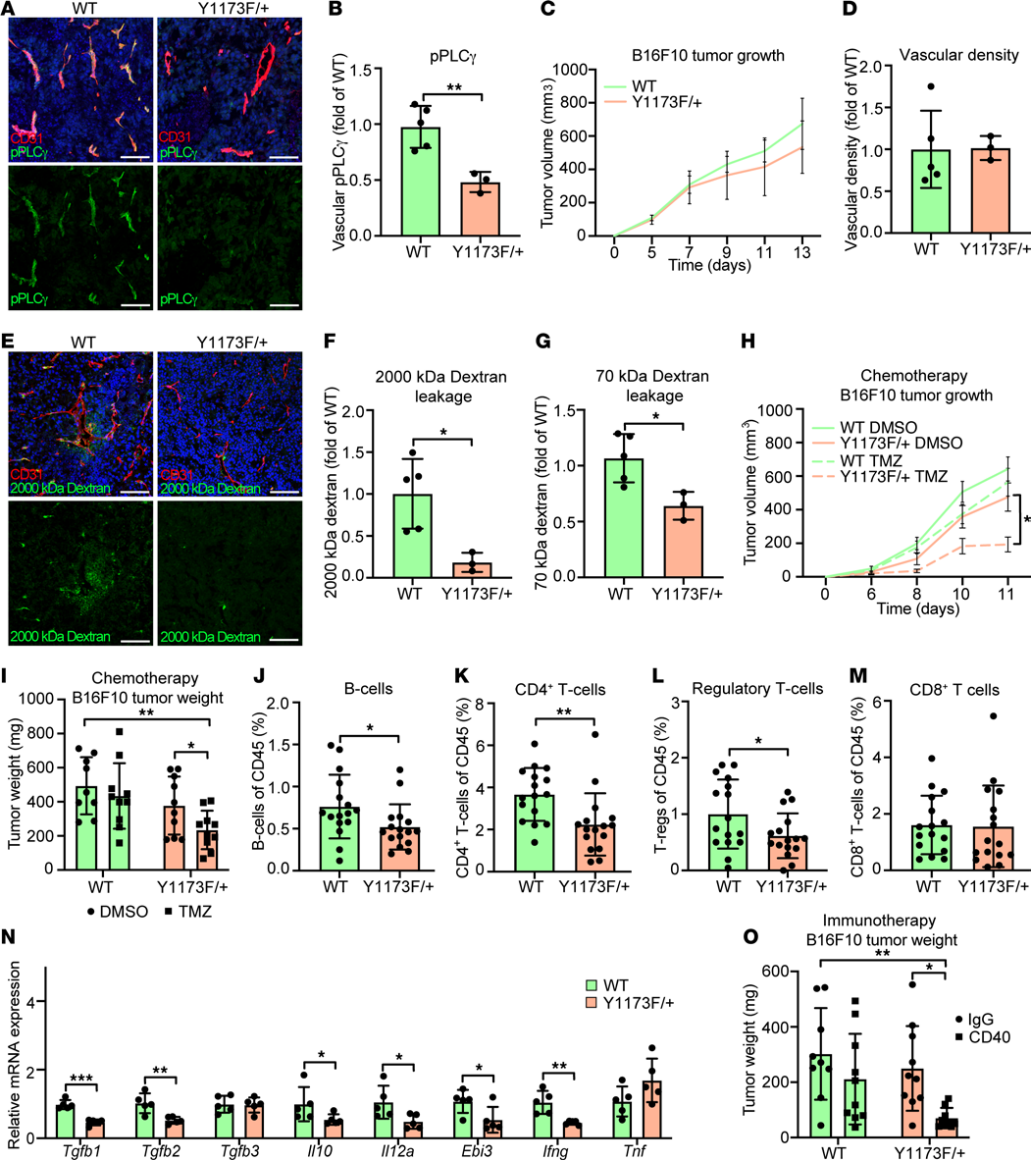
VEGFR2 pY1173/PLCγ signaling in experimental tumors affects vascular leakage, response to therapy, and antitumor immunity. (**A** and **B**) Immunostaining (**A**) and mean fluorescent intensity (MFI) quantification (**B**) of pPLCγ Y783 in *Vegfr2^+/+^* (WT) and *Vegfr2^Y1173F/+^* (Y1173F/+) B16F10 melanoma tumors; *n* = 5 (WT) and 3 (Y1173F/+) mice, ≥ 3 fields of view/experiment. Scale bar: 100 μm. Unpaired 2-tailed Students’ *t* test. (**C**) Tumor growth of WT and Y1173F/+ B16F10 tumors; *n* = 5 (WT) and 3 (Y1173F/+) mice. 1-way ANOVA. (**D**) Quantification of vessel density (MFI) from **A**; *n* = 5 (WT) and 3 (Y1173F/+) mice, ≥ 3 fields of view/tumor. Unpaired 2-tailed Students’ *t* test. (**E** and **F**) Representative image (**E**) and quantification (**F**) of extravasated fixable 2,000 kDa FITC-dextran in B16F10 tumors; *n* = 5 (WT) and 3 (Y1173F/+) mice, ≥ 3 fields of view/experiment. Scale bar:100 μm. Unpaired 2-tailed Students’ *t* test. (**G**) Fluorescent intensity of 70 kDa TRITC-dextran extracted from B16F10 tumors; *n* = 5 (WT) and 3 (Y1173F/+) mice. Unpaired 2-tailed Students’ *t* test. (**H** and **I**) Tumor growth (**H**) and tumor weights (**I**) of WT and Y1173F/+ B16F10 tumors treated with 5 mg/kg Temozolomide (TMZ) or DMSO control; *n* = 9–10 mice/group. 2-way ANOVA (**H**), 1-way ANOVA (**I**). (**J**–**M**) Percent B cells (CD19) (**J**), helper T cells (CD3 and CD4 costaining) (**K**), regulatory T cells (CD3, CD4, CD25, and Foxp3 costaining) (**L**), cytotoxic T cells (CD3 and CD8 costaining) (**M**) in WT and Y1173F/+ B16F10 tumors; *n* = 16/genotype. Unpaired 2-tailed Students’ *t* test. (**N**) mRNA expression of TGFβ (*Tgfb1-3*), IL10 (*Il10*), IL35 (*Il12a*, *Ebi3*), IFNγ (*Ifng*), and TNFα (*Tnf*) in WT and Y1173F/+ B16F10 tumors; *n* = 5 mice/genotype. Unpaired 2-tailed Students’ *t* test. (**O**) Tumor weight of CD40- or IgG-treated WT and Y1173F/+ B16F10 tumors harvested at day 12; *n* = 9–10 mice/group. 1-way ANOVA. Data represent mean ± SD or SEM (**H**). *P < 0.05, **P < 0.01, ***P < 0.001.

The clinical relevance of endothelial PLCγ expression in RCC prompted further analysis using experimental tumor models. Two subcutaneous tumor models, B16F10 melanoma and T241 fibrosarcoma, were inoculated in the flanks of WT mice and in mice in which one *Vegfr2* allele carried the Y1173F mutation. The heterozygous *Vegfr2^Y1173F/+^* mutants were born at the expected Mendelian ratios, whereas the homozygous *Vegfr2^Y1173F/Y1173F^* mutant pups were lost during embryogenesis in agreement with earlier reports (15). Isolated ECs (iECs) from *Vegfr2^Y1173F/+^* lung displayed a marked loss in pPLCγ levels in response to VEGFA compared with WT (Supplemental Figure 2, H and I), thus demonstrating the relevance of this mouse model. In agreement, the *Vegfr2^Y1173F/+^* B16F10 vasculature showed reduced levels of pPLCγ (Figure 2, A and B). Tumor growth rates and vascular density were similar when comparing WT with *Vegfr2^Y1173F/+^* mice inoculated with either B16F10 (Figure 2, C and D) or T241 fibrosarcoma (Supplemental Figure 2, J and K). The vasculature in solid tumors is characterized by hypoxia-driven VEGFA expression, a disrupted endothelial barrier, and elevated vascular leakage (1, 24). Vascular leakage in the B16F10 and T241 vasculature was determined by i.v. injection of FITC-conjugated 2,000 kDa fixable or TRITC-conjugated 70 kDa nonfixable dextran before tumor collection. B16F10 melanoma vessels in *Vegfr2^Y1173F/+^* mice showed reduced dextran leakage compared with WT mice, assessed by quantifying fixed dextran on sections and by extraction of nonfixable dextran (Figure 2, E-G). Tumor vessel leakage was similarly suppressed in T241 fibrosarcoma in *Vegfr2^Y1173F/+^* mice (Supplemental Figure 2, L–N). The stabilized B16F10 tumor vasculature in the *Vegfr2^Y1173F/+^* mice conferred increased sensitivity of these tumors to chemotherapy; treatment of *Vegfr2^Y1173F/+^* mice with a low dose temozolomide (TMZ, 5 mg/kg) suppressed B16F10 melanoma growth while tumors in WT mice remained unaffected (Figure 2, H and I).

The enhanced immune activation in the RCC *PLCG1*-low subgroup implied a potential role for vascular VEGFR2 Y1173/PLCγ signaling in immune cell infiltration and activation. Profiling of immune cells in the B16F10 tumors showed reduced numbers of B cells (CD19^+^) and helper T cells (CD3^+^ CD4^+^) as well as Tregs (CD3^+^ CD4^+^ CD25^+^ Foxp3^+^) in *Vegfr2^Y1173F/+^* tumors compared with WT tumors (Figure 2, J–L). Notably, the overall number of immune cells (CD45^+^) did not differ between genotypes and other immune cell populations, including cytotoxic T cells (CD3^+^ CD8^+^), dendritic cells (CD11c^+^ MHC II^hi^), and macrophages (CD11b^+^ F4/80^+^) were present at similar levels in B16F10 tumors from WT and Y1173F mice (Figure 2M and Supplemental Figure 2, O–Q). In the human KIRC cohort, *PLCG1* expression correlated with mRNA expression of the B cell markers CD19 and CD20 and the Treg marker Foxp3, but not with the T cell marker CD8 (Supplemental Table 2). This human RCC data is in agreement with the higher prevalence of B cells and Tregs in the mouse WT B16F10 tumors, which has abundant endothelial PLCγ signaling (Figure 2, A and J–M).

Quantitative PCR (qPCR) of whole tumor lysate for transcript levels of the cytokines TGFβ (*Tgfb1–2*), IL10 (*Il10*), and IL35 (*Ebi3* and *Il12a*), associated with suppressed antitumor immunity (25), showed reduced levels in *Vegfr2^Y1173F/+^* tumors (Figure 2N). *Ebi3* can also dimerize with *Il27* to form IL27, and *Il12a* with *Il12b* to form IL12; both *Il27* and *Il12b* were expressed at low and comparable levels in the B16F10 tumors from WT and *Vegfr2^Y1173F/+^* mice (Supplemental Figure 2R). The inflammatory cytokine IFNγ (*Ifng*), assigned a pleiothropic role in tumor immunity (26), was also reduced in *Vegfr2^Y1173F/+^* tumors, whereas the levels of *Tgfb3* and *Tnfa* remained unaffected (Figure 2N). In line with these data, in the KIRC cohort, *PLCG1* transcripts covaried with expression of *TGFB1–3* and *IL12A* (Supplemental Table 3). Overall, these data suggest that tumor vascular VEGFR2 pY1173/PLCγ signaling may steer an immune-suppressive microenvironment. Therefore, the response to immunotherapy treatment with CD40 agonistic antibodies was tested. CD40 agonistic antibodies enhance antitumor immunity by increasing antigen presentation through CD40, expressed on antigen-presenting cells (APCs), including DC and B cells, which promotes activation and expansion of cytotoxic T cells (27). Treatment of *Vegfr2^Y1173F/+^* mice with anti-CD40 antibodies suppressed growth of B16F10 tumors, while WT mouse tumors were resistant to this therapy (Figure 2O). These data indicate that the extent of PLCγ signaling in tumor vessels correlates with the anti-tumor immune status and the response to immunotherapy.

### VEGFR2 pY1173 regulates VEGFA-induced vascular permeability.

**Figure 3:**
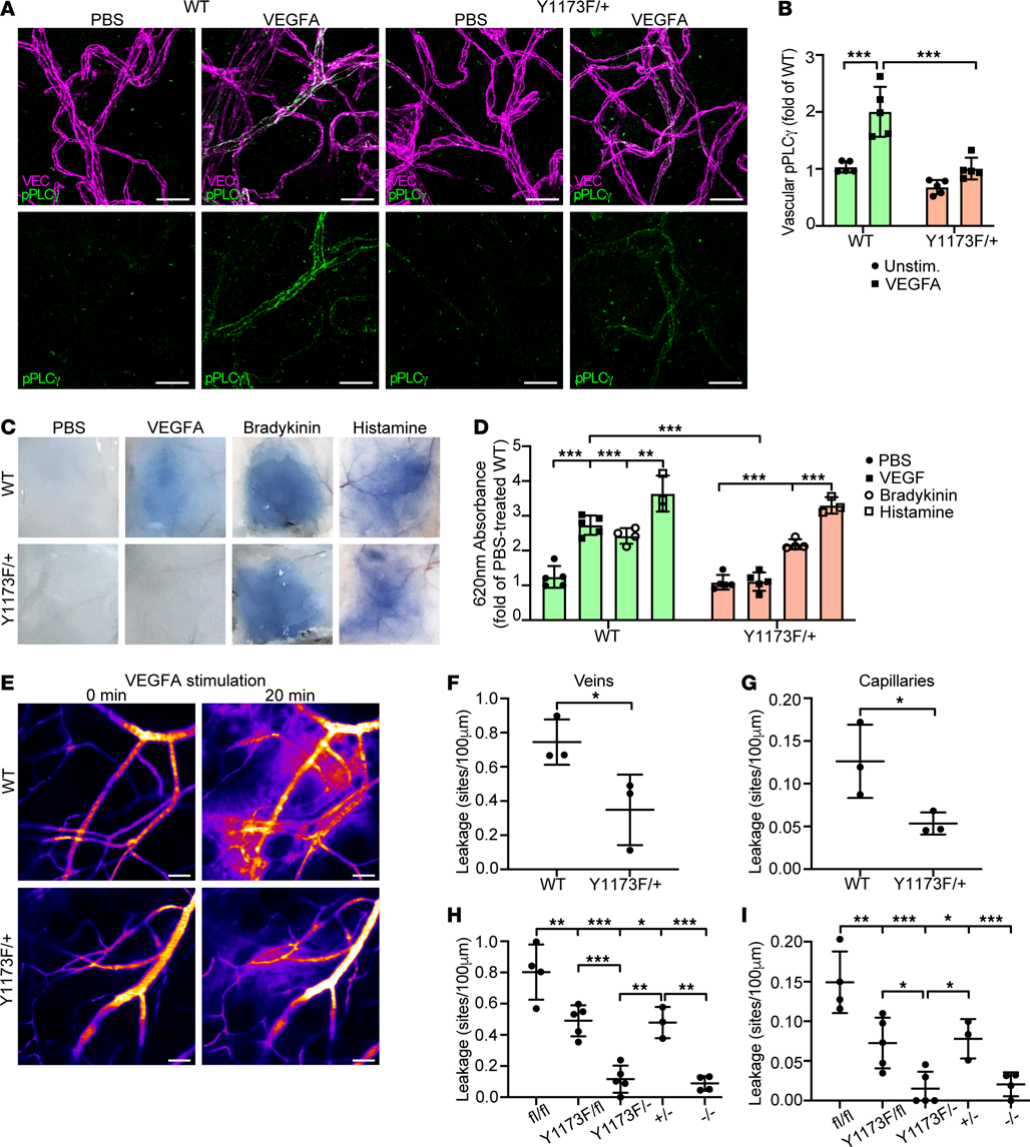
VEGFR2 pY1173 signaling regulates VEGFA-induced vascular permeability in the healthy skin. (**A**) Representative images of immunofluorescent staining for VE-cadherin (VEC) and pPLCγ Y783 in the back skin of *Vegfr2^+/+^* WT and *Vegfr2^Y1173F/+^* heterozygous (Y1173F/+) mice, subsequent to intradermal injection of PBS or VEGFA. Scale bar: 20 μm. (**B**) Quantification of mean fluorescent intensity of vascular pPLCγ Y783 from **A**; *n* = 5 mice/genotype, ≥ 2 fields of view/mouse. 1-way ANOVA. (**C**) Representative images of Evans blue leakage in response to PBS, VEGFA, bradykinin, or histamine in the back skin of WT and Y1173F/+ mice. (**D**) Quantification (620 nm absorbance) of extravasated Evans blue from **C**. Values are shown as fold of PBS-treated control, normalized to tissue weight; *n* = 3–5 mice/group. 1-way ANOVA. (**E**) Representative time-lapse image of VEGFA-induced vascular permeability of 2,000 kDa FITC dextran in the ear dermis of WT and Y1173F/+ mice. Scale bar: 50 μm. (**F** and **G**) Number of leakage sites/100 μm in WT and *Vegfr2^Y1173F/+^* mice, in veins (**F**) and capillaries (**G**); *n* = 3 mice/genotype, ≥ 2 fields of view/mouse. Unpaired 2-tailed Students’ *t* test. (**H** and **I**) Ear dermis leakage in response to VEGFA in tamoxifen-treated *Vegfr2^fl/fl^; Cdh5-Cre^–^* (fl/fl)*, Vegfr2^Y1173F/fl^; Cdh5-Cre^–^* (Y1173F/fl), *Vegfr2^Y1173F/fl^; Cdh5-Cre^+^* (Y1173F/*–*), *Vegfr^+/fl^; Cdh5-Cre^+^* (+/*–*), *Vegfr2^fl/fl^; Cdh5-Cre^+^* (*–*/*–*)*,* quantified as leakage sites/100 μm, in veins (**H**) and capillaries (**I**); *n* = 3–5 mice/genotype, ≥ 2 fields of view/mouse. 1-way ANOVA. Data represent mean ± SD. *P < 0.05, **P < 0.01, ***P < 0.001.

To examine the role of PLCγ signaling downstream of VEGFA-induced pY1173 in vivo in the healthy endothelium, *Vegfr2^Y1173F/+^* and WT mice were injected intradermally with VEGFA or PBS in the back skin. Immunofluorescent staining showed reduced levels of VEGFA-induced pPLCγ Y783 in the endothelium of *Vegfr2*^Y1173F/+^ mice compared with WT mice, supporting the requirement of pY1173 signaling for PLCγ activation in vivo (Figure 3, A and B).

The importance of pY1173 signaling in regulation of the vascular barrier in normal vessels was assessed by analysis of intradermal leakage of the albumin-bound colloidal dye Evans blue after administration of VEGFA in the back skin. Leakage increased 2.7-fold in the WT dermis, while the effect of VEGFA was essentially obliterated in *Vegfr2^Y1173F/+^* mice (Figure 3, C and D). In contrast, administration of the inflammatory cytokines bradykinin or histamine elicited similar leakage responses in WT and *Vegfr2^Y1173F/+^* mice (Figure 3, C and D). Live imaging of vascular leakage using sensitive, noninvasive real-time imaging to determine its kinetics (28) confirmed a significant suppression of VEGFA-mediated vascular leakage in the *Vegfr2^Y1173F/+^* dermal vasculature, quantified as number of leakage sites per vessel length (Figure 3, E–G). Leakage from dermal venules as well as capillaries was reduced by approximately 50%, in accordance with the Y1173F heterozygosity.

To confirm the impact of pY1173/PLCγ signaling on vascular permeability and circumvent the embryonic lethality of *Vegfr2^Y1173F/Y1173F^* mice, we bred *Vegfr2^Y1173F/+^* heterozygous mice onto a *Vegfr2^fl/fl^; Cdh5-CreERT2* background. Tamoxifen-mediated excision of the WT *Vegfr2* allele generated *Vegfr2^Y1173F/–^* mice expressing only one *Vegfr2* allele in endothelial cells, carrying the Y1173F mutation. The efficiency of VEGFR2 downregulation in tamoxifen-treated *Vegfr2^Y1173F/–^* mice was validated by anti-VEGFR2 immunostaining (Supplemental Figure 3, A and B).

The effect of elimination of pY1173-signaling in the *Vegfr2^Y1173F/–^* model was examined by assessing vascular density in the ear dermis of 8-week-old mice. Thirty days after the initiation of tamoxifen treatment, there was no difference in the vascular density between WT, *Vegfr2^Y1173F/–^,* and *Vegfr2^Y1173F/fl^* mice (expressing one Y1173F mutant *Vegfr2* allele and one WT allele) (Supplemental Figure 3, C and D), suggesting that pY1173 signaling is nonessential for endothelial maintenance in the adult vascular bed, although organ-dependent effects cannot be excluded. However, VEGFA-induced leakage in the ear dermis was nearly completely suppressed in *Vegfr2^Y1173F/–^* mice compared with *Vegfr2^+/–^* mice (expressing only one *Vegfr2* WT allele in endothelial cells) or *Vegfr2^Y1173F/fl^* mice (Figure 3, H and I). Interestingly, the suppression of VEGFA-induced leakage was similar to that seen after endothelial removal of both *Vegfr2* alleles, demonstrating the requirement for pY1173 signaling for transient destabilization of endothelial junctions (Figure 3, H and I).

The vascular barrier is composed of endothelial cells, the surrounding mural support, and the basement membrane. Analysis of the skin vasculature of *Vegfr2^Y1173F/+^* mice revealed similar vessel density, pericyte coverage, levels of basement membrane and junctional markers as seen in the WT *Vegfr2^+/+^* vasculature (Supplemental Figure 3, E–J). Moreover, unprovoked, basal dermal leakage measured in mice with systemic circulation of fluorescent dextran or Evans blue, was similar between WT and *Vegfr2*^Y1173F/+^ mice (Supplemental Figure 3, K–N), indicating that the stability of the vascular barrier in the absence of leakage agonists was unaffected by loss of pY1173 signaling.

We conclude that dermal vessel permeability and leakage of macromolecules in vivo depends on signaling downstream of the VEGFR2 Y1173 phosphosite.

### PLCγ signaling destabilizes the endothelial cell barrier.

**Figure 4:**
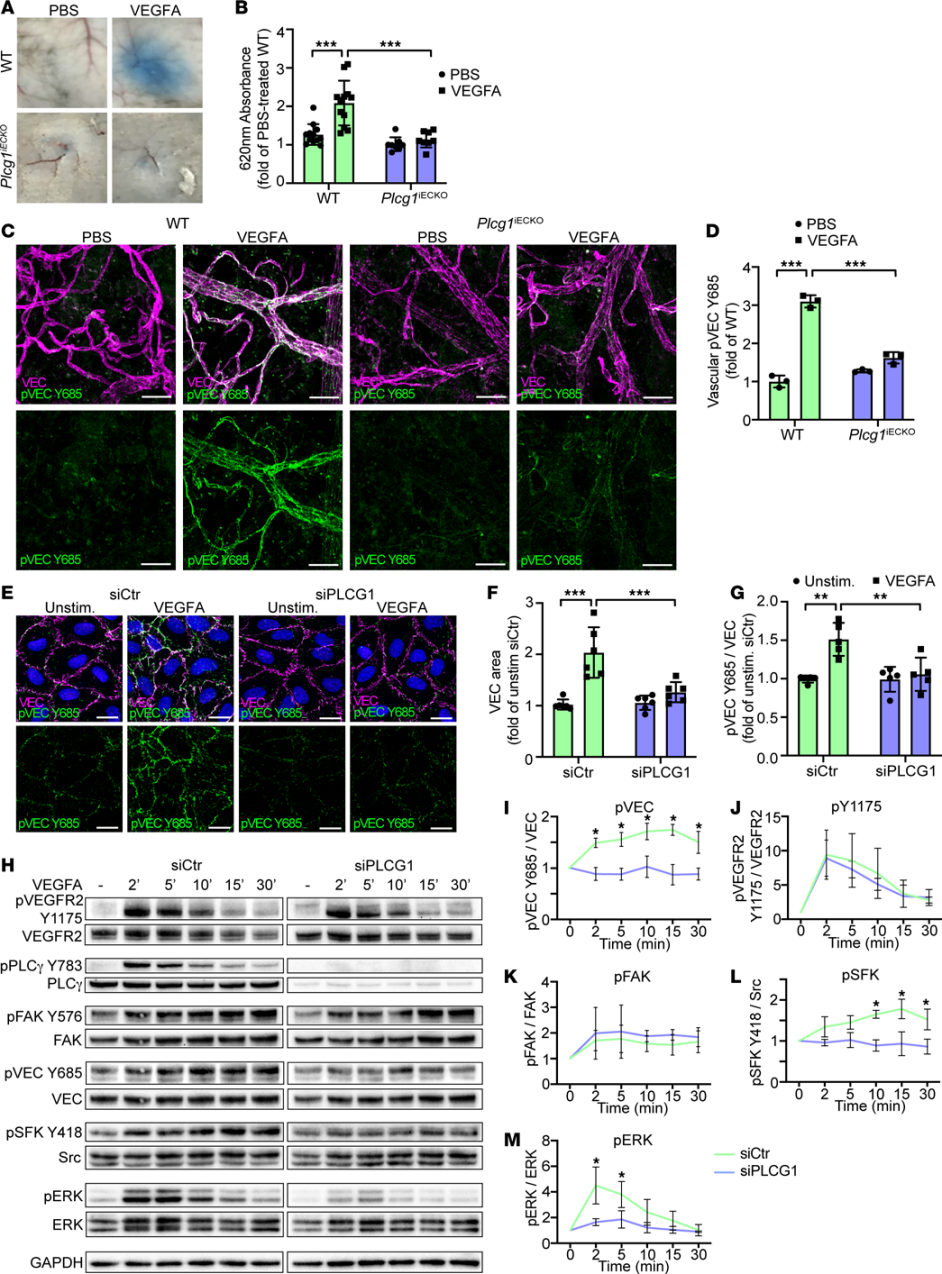
pY1173/PLCγ signaling leads to adherens junction disruption and phosphorylation. (**A**) Evans blue leakage in the back skin in response to PBS or VEGFA in tamoxifen-treated *Plcg1^fl/fl^; Cdh5-Cre –* (WT), and *Plcg1^fl/fl^; Cdh5-Cre +* (*Plcg1^iECKO^*) mice. (**B**) Quantification of extravasated Evans blue from **A** shown as fold of PBS-treated WT mice, normalized to tissue weight; *n* = 12 (WT), and 8 (*Plcg1^iECKO^*) mice. 1-way ANOVA. (**C**) Immunofluorescent staining with antibodies against VE-cadherin (VEC) and pVEC Y685 in the back skin of tamoxifen-treated WT and *Plcg1^iECKO^* mice after intradermal injection of PBS or VEGFA. Scale bar: 20 μm. (**D**) Quantification of mean fluorescent intensity for vascular pVEC Y685 from **C**; *n* = 3 mice/genotype, ≥ 2 fields of view/mouse. 1-way ANOVA. (**E**) Immunofluorescent staining of VEC and pVEC Y685 of unstimulated or VEGFA-stimulated HUVECs (100 ng/mL, 5 min), silenced for *PLCG1* (*siPLCG1*) or treated with control siRNA (*siCtr*). Nuclei stained with DAPI (blue). Scale bar: 20 μm. (**F** and **G**) Quantification of VEC area (**F**); *n* = 6 independent experiments, ≥ 3 fields of view/experiment and pVEC Y685 levels (**G**); *n* = 5 independent experiments, ≥ 3 fields of view/experiment from **E**. 1-way ANOVA. (**H**) Representative Western blot showing downstream VEGFA-activated signaling in *siCtr* or *siPLCG1*-treated HUVECs. (**I**–**M**) Quantification of at least 3 independent experiments from **H**, for pVEC Y685 (**I**), pVEGFR2 Y1175 (**J**), pFAK (**K**), pSFK Y418 (**L**), and pERK (**M**), shown as fold change of unstimulated samples. 1-way ANOVA. Data represent mean ± SD. *P < 0.05, **P < 0.01, ***P < 0.001.

To pinpoint the contribution of PLCγ in the adult endothelium in vivo, *Plcg1^fl/fl^* mice (29) were crossed with the *Cdh5-CreERT2* strain to generate *Plcg1^fl/fl^; Cdh5-CreERT2* mice. Cre-positive *Plcg1^fl/fl^* mice*,* lacking expression of PLCγ specifically in ECs after tamoxifen administration, are referred to as *Plcg1^iECKO^* mice (see Supplemental Figure 4, A and B for floxing efficiency in lung iECs). *Plcg1^iECKO^* mice failed to respond to intradermal VEGFA administration with increased Evans blue extravasation, relative to cre-negative *Plcg1^fl/fl^* (WT) mice, demonstrating an essential role for endothelial PLCγ in regulation of vascular leakage in vivo (Figure 4, A and B).

Regulation of vascular permeability by modulating endothelial adherens junction stability is a key in vivo response downstream of VEGFA/VEGFR2 activation. VE-cadherin is essential in stabilizing endothelial adherens junctions by forming homophilic complexes between ECs (30). Phosphorylation of VE-cadherin at Y685 by SFKs is accompanied by VE-cadherin internalization and thereby disruption of the homophilic interactions (31). In this process, the morphology of VE-cadherin junctions transition from linear to broad, jagged patterns in vitro. The transition of VE-cadherin morphology to a jagged phenotype at VEGFA-induced vascular leakage sites was confirmed in vivo, in dermal vessels (Supplemental Figure 4C). An increased VE-cadherin area, marking junction disruption when treated with VEGFA, was also displayed in lung iECs from WT mice. In contrast, junctions remained linear in cultures of iECs from *Vegfr2^Y1173F/+^* mice (Supplemental Figure 4, D and E). Moreover, VE-cadherin pY685 levels increased in vivo in the WT mouse back skin dermis after injection of VEGFA, but remained low in *Vegf-r2^Y1173F/+^* mice (Supplemental Figure 4, F and G) and *Plcg1^iECKO^* mice (Figure 4, C and D). VEGFA-induced VE-cadherin disruption and phosphorylation at Y685 were also suppressed in HUVECs after siRNA-mediated silencing of *PLCG1,* assessed by immunostaining and immunoblotting (Figure 4, E–I). *PLCG1* silencing still allowed VEGFA-induced phosphorylation of VEGFR2 Y1175 and focal adhesion kinase (FAK) (Figure 4, H, J, and K), however, activation of SFKs and ERK1/2 were suppressed to baseline in *siPLCG1*-treated HUVECs (Figure 4, H, L, and M). VEGFA-induced SFK- and ERK1/2-signaling was also suppressed in iECs from *Vegfr2^Y1173F/+^* mice compared with WT mice, while pY949 levels were unaffected (Supplemental Figure 4, H–L). Therefore, VEGFR2 pY1173/PLCγ signaling was required for VEGFA-mediated SFK and ERK1/2 activation, phosphorylation of VE-cadherin at Y685, and disruption of endothelial adherens junctions.

### PLCγ induction of vascular permeability requires eNOS activation.

**Figure 5:**
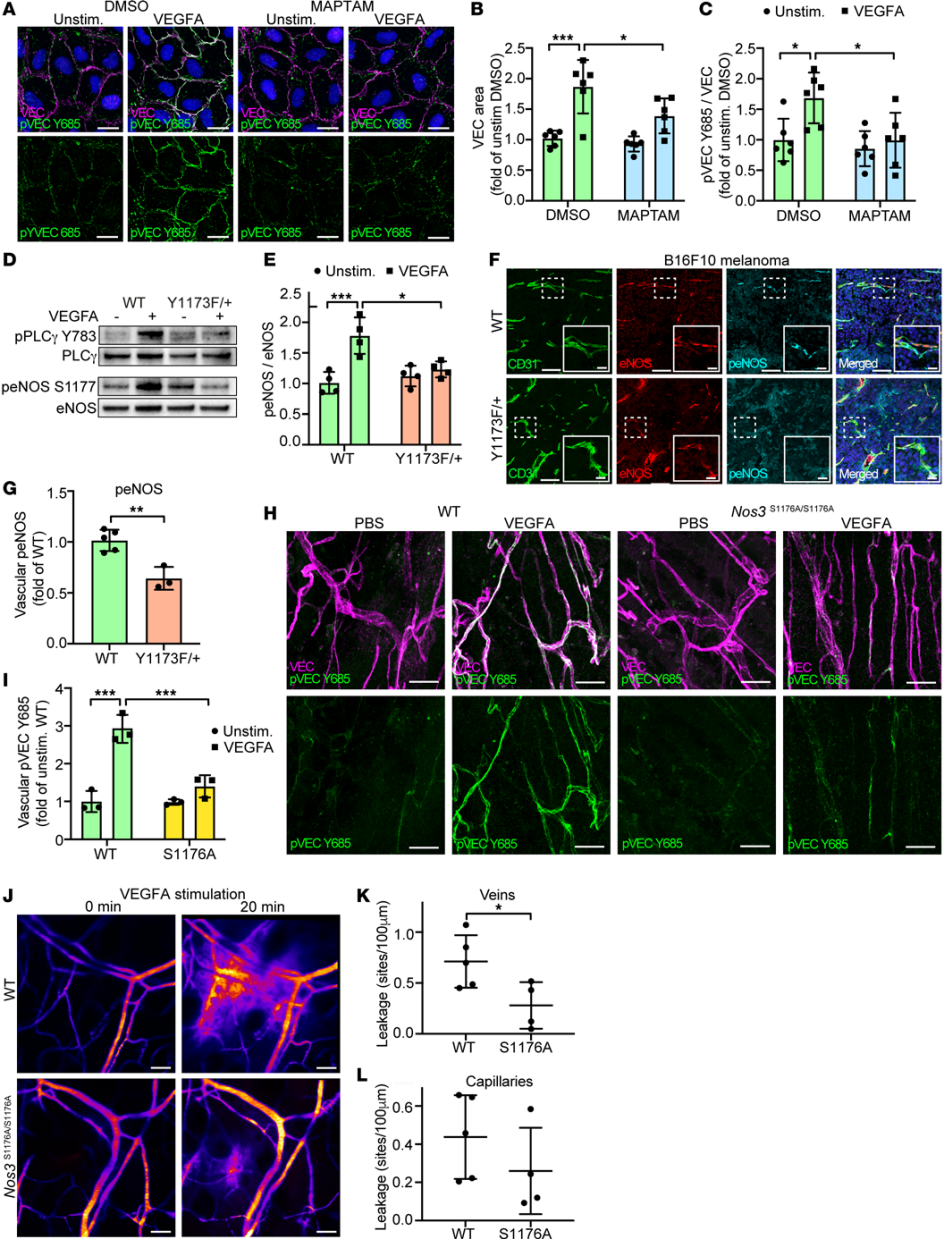
PLCγ activates eNOS in a Ca^2+^-dependent manner to induce VEGF-dependent vascular leakage. (**A**) Representative VE-cadherin (VEC) and pVEC Y685 immunostainings of HUVECs pretreated for 15 minutes with DMSO (control) or 5 μM MAPTAM prior to VEGFA stimulation (100 ng/ml, 5 min). Nuclei stained with DAPI (blue). Scale bar: 20 μm. (**B** and **C**) Quantification of VEC area (**B**) and pVEC Y685 levels (**C**) from **A**; *n* = 6 independent experiments, ≥ 3 fields of view/experiment. 1-way ANOVA. (**D** and **E**) Representative Western blot (**D**) and quantification (**E**) of peNOS S1177 levels in VEGFA-stimulated isolated lung ECs from WT and *Vegfr2^Y1173F/+^* (Y1173F/+) mice; *n* = 4 mice/genotype. 1-way ANOVA. (**F** and **G**) Representative immunostaining (**F**) and mean fluorescent intensity (MFI) quantification (**G**) of eNOS and peNOS S1176 in B16F10 melanomas from *Vegfr2^+/+^* (WT) and *Vegfr2^Y1173F/+^* (Y1173F/+) mice; *n* = 5 (WT) and 3 (Y1173F/+) mice, ≥ 2 fields of view/tumor. Scale bar: 100 μm. Scale bar inset: 20 μm. Unpaired 2-tailed Students’ *t* test. (**H**) Immunofluorescent staining with antibodies against VEC and pVEC Y685 in the back skin of *NOS3^+/+^* (WT) and *Nos3^S1176A/S1167A^* (S1176A) mice after intradermal injection of PBS or VEGFA. Scale bar: 20 μm. (**I**) Quantification of MFI values for vascular pVEC Y685 from **H**; *n* = 3 mice/genotype, ≥ 2 fields of view/mouse. 1-way ANOVA. (**J**) Representative time-lapse image of VEGFA-stimulated vascular extravasation of a 2,000 kDa FITC-dextran in the ear dermis of WT and S1176A mice. Scale bar: 50 μm. (**K** and **L**) Number of leakage sites/100 μm in WT and S1176A mice in ear dermis veins (**K**) and capillaries (**L**); *n* = 4–5 mice/genotype, ≥ 2 fields of view/mouse. Unpaired 2-tailed Students’ *t* test. Data represent mean ± SD. *P < 0.05, **P < 0.01, ***P < 0.001.

VEGFA provokes a rapid release of Ca^2+^ from intracellular stores by activation of PLCγ (17). Disruption of adherens junctions is known to involve Ca^2+^ signaling (32), and, therefore, we studied the effect of removal of intracellular Ca^2+^ using the cell-permeable Ca^2+^ chelator MAPTAM, or by inhibiting PLCγ-induced Ca^2+^ fluxes using the PLCγ inhibitor U73122 (Supplemental Figure 5A). MAPTAM-treated HUVECs showed reduced VE-cadherin disruption, estimated as a change in area, as well as reduction of VE-cadherin Y685 phosphorylation after VEGFA-stimulation (Figure 5, A–C). eNOS depends on Ca^2+^ for its effects on a broad range of endothelial processes induced by VEGFR2-signaling (33). Moreover, both activation of SFKs and induction of VE-cadherin pY685 are suppressed in mice expressing a catalytically inactive eNOS mutant, challenged with oxygen-induced retinopathy (34). In HUVECs silenced for *PLCG1* or, alternatively, treated with the PLCγ inhibitor U73122 (Supplemental Figure 5A), VEGFA-mediated eNOS activation was nearly extinguished, whereas pY1175 phosphorylation remained intact (Supplemental Figure 5, A–G). Furthermore, activation of eNOS was suppressed in VEGFA-stimulated iECs from *Vegfr2^Y1173F/+^* mice, relative to iECs from WT mice (Figure 5, D and E), as well as in tumor vessels in B16F10 melanoma growing in *Vegfr2 ^Y1173F/+^* mice (Figure 5, F and G). In accordance with an important role for eNOS activity in VEGFA-induced junction disintegration, VEGFA-induced VE-cadherin pY685 in dermal vessels (Figure 5, H and I) and intradermal leakage of dextran (Figure 5, J–L) were markedly suppressed in *Nos3^S1176A/S1176A^* mice, homozygous for an inactivating mutation at S1176, required for eNOS catalytic activity.

### Ca^2+^ release rescues activation of Src and VE-cadherin pY685 accumulation upon loss of PLCγ signaling.

**Figure 6:**
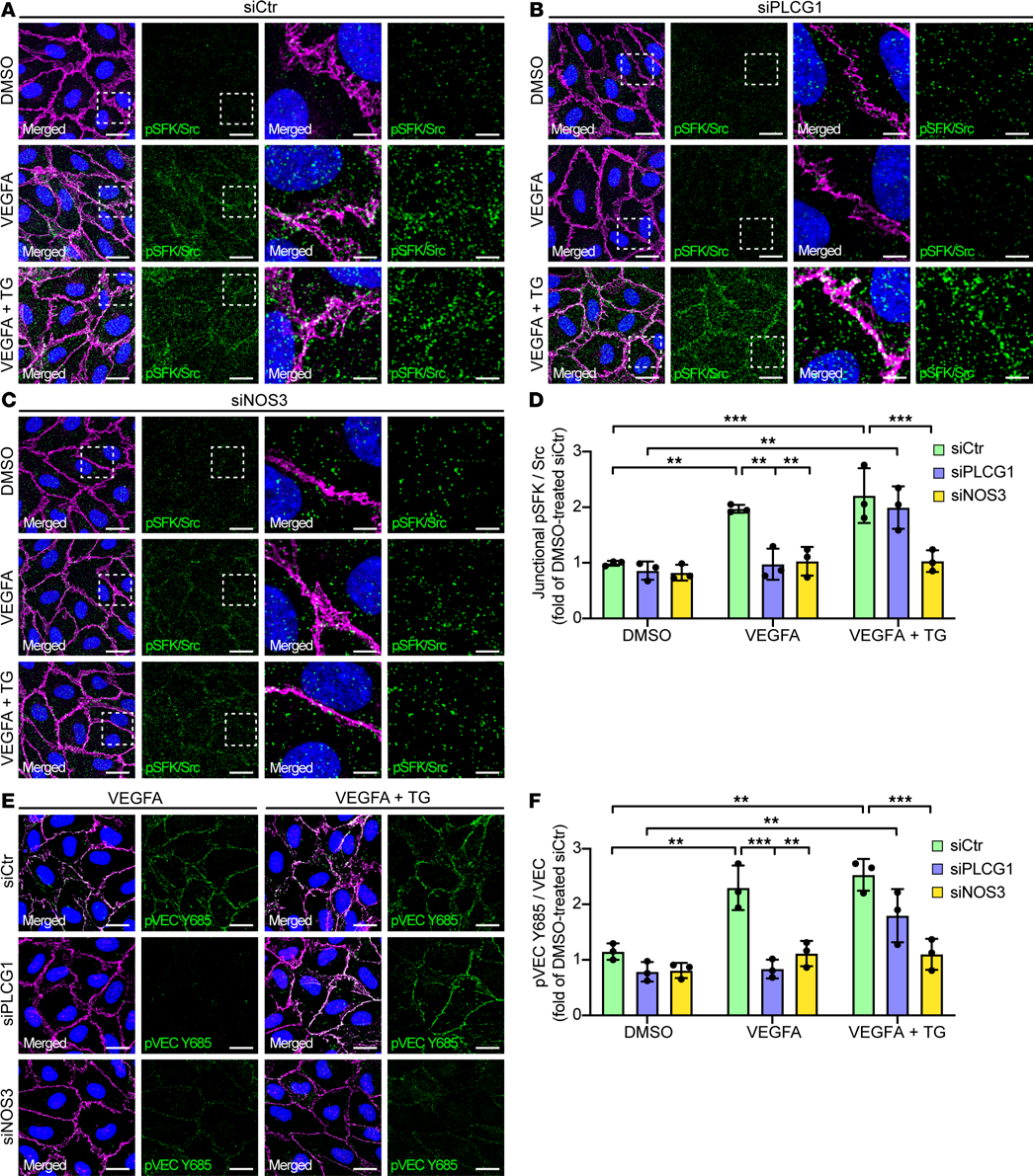
TG rescues activation of Src and VE-cadherin phosphorylation in response to VEGFA, after removal of PLCγ. (**A**–**C**) PLA using antibodies against Src and pSFK Y418, visualizing phosphorylation of Src on Y418, in HUVECs treated for 5 min with DMSO, 100 ng/ml VEGFA or 100 ng/ml VEGFA + 1uM TG, and pretreated with *siCtr* (**A**), *siPLCG1* (**B**), or *siNOS3* (**C**). Junctions are stained for VE-cadherin (VEC) and nuclei with DAPI (blue). Scale bar: 20 μm. Boxed regions in left panels are magnified in panels to the right. Scale bar: 5 μm. (**D**) MFI quantifications of junctional PLA signals representing Src phosphorylated on Y418 from **A**–**C**; *n* = 3 independent experiments, ≥ 3 fields of view/experiment. 1-way ANOVA. (**E**) Representative images of immunofluorescent stainings with antibodies against VEC and pVEC Y685, of HUVECs treated with VEGFA or VEGFA+TG, pretreated with *siCtr*, *siPLCG1* or *siNOS3*. Scale bar: 20 μm. (**F**) MFI quantification of data from **E** shown as fold of DMSO-treated *siCtr*; *n* = 3 independent experiments, ≥ 3 fields of view/experiment. 1-way ANOVA. Data represent mean ± SD. *P < 0.05, **P < 0.01, ***P < 0.001.

To delineate the molecular mechanism in eNOS-regulated vascular permeability and, in particular, the role of SFK activation, expression of either eNOS or Src was silenced in endothelial cells. Downregulation of eNOS resulted in elevated basal and reduced induction of SFK pY418 in response to VEGFA stimulation, whereas downregulation of Src left eNOS activation unaffected (Supplemental Figure 6, A–D), placing Src activation downstream of eNOS. Of the SFK members, Src has previously been shown to be critical in VEGF-A-, as well as bradykinin-induced vascular permeability (31, 35). However, antibodies against SFK pY418 recognize a sequence that is identical between Src, Yes, and Fyn. To overcome the challenge of reagents cross-reacting between different SFKs, a proximity ligation assay (PLA) was employed, using oligonucleotide-conjugated antibodies detecting Src or Yes in combination with antibodies detecting pSFK Y418. As seen in Figure 6, A–D, VEGF-A stimulation of HUVECs enhanced the number of junction-associated Src/pSFK signals only in cells with functional PLCγ- and eNOS-signaling (Figure 6, A–D). In contrast, the presence of activated Yes at endothelial junctions was already high before VEGFA treatment, and junctional pSFK/Yes PLA signals were unaffected by downregulation of eNOS (Supplemental Figure 6, E and F). Thus, eNOS regulates activation of Src but not Yes.

The crucial involvement of Ca^2+^ was further revealed by rescue experiments using Thapsigargin (TG) to inhibit the sarcoendoplasmatic/endoplasmic reticulum Ca^2+^ ATPase (SERCA), required for depletion of Ca^2+^ from the cytosol (36). TG treatment of HUVECs enhanced cytosolic Ca^2+^ levels, independent of PLCγ activation in cells treated with the PLCγ inhibitor U73122 or DMSO control (Supplemental Figure 6G). Notably, addition of TG rescued VEGFA-stimulated Src activation (Figure 6, A–D) and VE-cadherin pY685 levels after siRNA-mediated downregulation of PLCγ, but not after downregulation of eNOS (Figure 6, E and F). TG treatment also rescued VEGFA-induced VE-cadherin pY685 levels subsequent to pharmacological inhibition of PLCγ (Supplemental Figure 6, H and I). These data show that restoring intracellular Ca^2+^ levels through TG treatment overcomes the loss of PLCγ-mediated Ca^2+^ release required for activation of eNOS and downstream phosphorylation of Src and VE-cadherin. In contrast, TG-mediated Ca^2+^ restoration could not compensate for the loss of eNOS, whose enzymatic activity is required for accumulation of Src pY418 and VE-cadherin pY685. This data further support Ca^2+^-dependent functions of eNOS, downstream of PLCγ, in regulation of endothelial junction stability.

Collectively, Ca^2+^ signaling induced by PLCγ is crucial for VEGFA-mediated vascular permeability by activating an eNOS/Src/VE-cadherin–signaling cascade.

### PKC-dependent eNOS activation mediates tyrosine nitration of Src, required for VEGF-induced vascular permeability.

**Figure 7:**
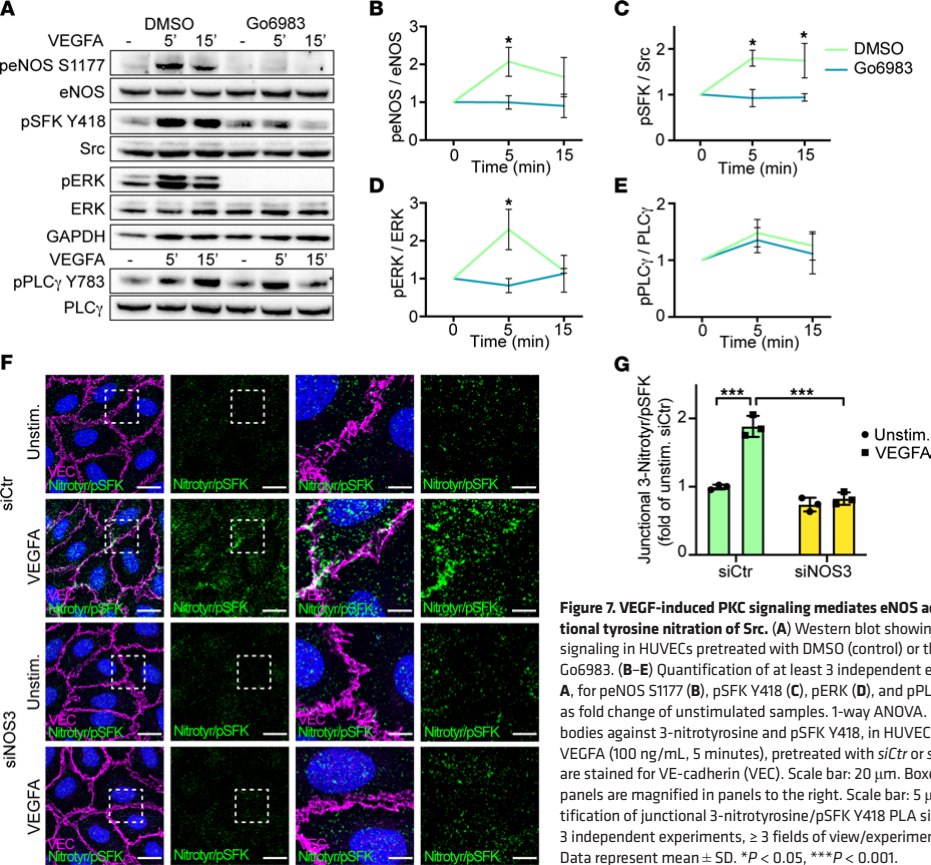
VEGF-induced PKC signaling mediates eNOS activation and junctional tyrosine nitration of Src. (**A**) Western blot showing VEGFA-activated signaling in HUVECs pretreated with DMSO (control) or the PKC inhibitor Go6983. (**B**–**E**) Quantification of at least 3 independent experiments from **A**, for peNOS S1177 (**B**), pSFK Y418 (**C**), pERK (**D**), and pPLCγ Y783 (**E**), shown as fold change of unstimulated samples. 1-way ANOVA. (**F**) PLA using antibodies against 3-nitrotyrosine and pSFK Y418, in HUVECs stimulated with VEGFA (100 ng/mL, 5 minutes), pretreated with *siCtr* or *siNOS3*. Junctions are stained for VE-cadherin (VEC). Scale bar: 20 μm. Boxed regions in left panels are magnified in panels to the right. Scale bar: 5 μm. (**G**) MFI quantification of junctional 3-nitrotyrosine/pSFK Y418 PLA signals from **F**; *n* = 3 independent experiments, ≥ 3 fields of view/experiment. 1-way ANOVA. Data represent mean ± SD. *P < 0.05, ***P < 0.001.

Several kinases have been described to phosphorylate eNOS at S1177, leading to subsequent enzymatic activation, including AKT (37–39), CAMK (40), AMPK (41), and PKCα (42). In an attempt to identify the responsible kinase for eNOS S1177 phosphorylation downstream of pY1173/PLCγ signaling, we used pharmacological inhibitors to block AKT — with Wortmannin, a PI3K inhibitor — ERK — with U0126, a MEK inhibitor — or PKC activation — with Go6983, a PKC inhibitor. VEGFA-induced activation of eNOS was unperturbed by inhibition of either AKT or ERK signaling (Supplemental Figure 7, A–F). In accordance, downstream SFK activation was unaffected by these inhibitors (Supplemental Figure 7, A–F), and VEGFA-induced phosphorylation of VE-cadherin Y685 still remained after treating HUVECs with Wortmannin or U0126 (Supplemental Figure 7, G–I). In contrast, inhibition of PKC using Go6983 efficiently hindered phosphorylation of eNOS in response to VEGFA (Figure 7, A and B), suggesting the involvement of one or several isoforms of PKC in eNOS activation. VEGFA-induced phosphorylation of Src and ERK was also reduced by PKC inhibition, whereas upstream PLCγ phosphorylation was unaffected (Figure 7, C–E).

eNOS activation and NO release have previously been shown to impact vascular permeability through posttranslational modification of cysteine and tyrosine residues in Src and other mediators (43–46). Src activity is known to be regulated by posttranslational modification of tyrosine residues through phosphorylation, which spurred our interest in tyrosine nitration. To investigate if pY1173/PLCγ signaling enhances modification of Src by tyrosine nitration in an eNOS dependent fashion, PLA was employed, using antibodies against 3-Nitrotyrosine, combined with antibodies against Src or pSFK. VEGFA-enhanced tyrosine nitration of Src was detected only in the presence of eNOS expression (Figure 7, F and G, and Supplemental Figure 7, J–L). Nitrated Src molecules localized both to the cytoplasmic compartment and to endothelial junctions (Supplemental Figure 7, J–L), whereas nitrated pSFK molecules showed a preferential junctional localization (Figure 7, F and G) in response to VEGFA.

**Figure 8:**
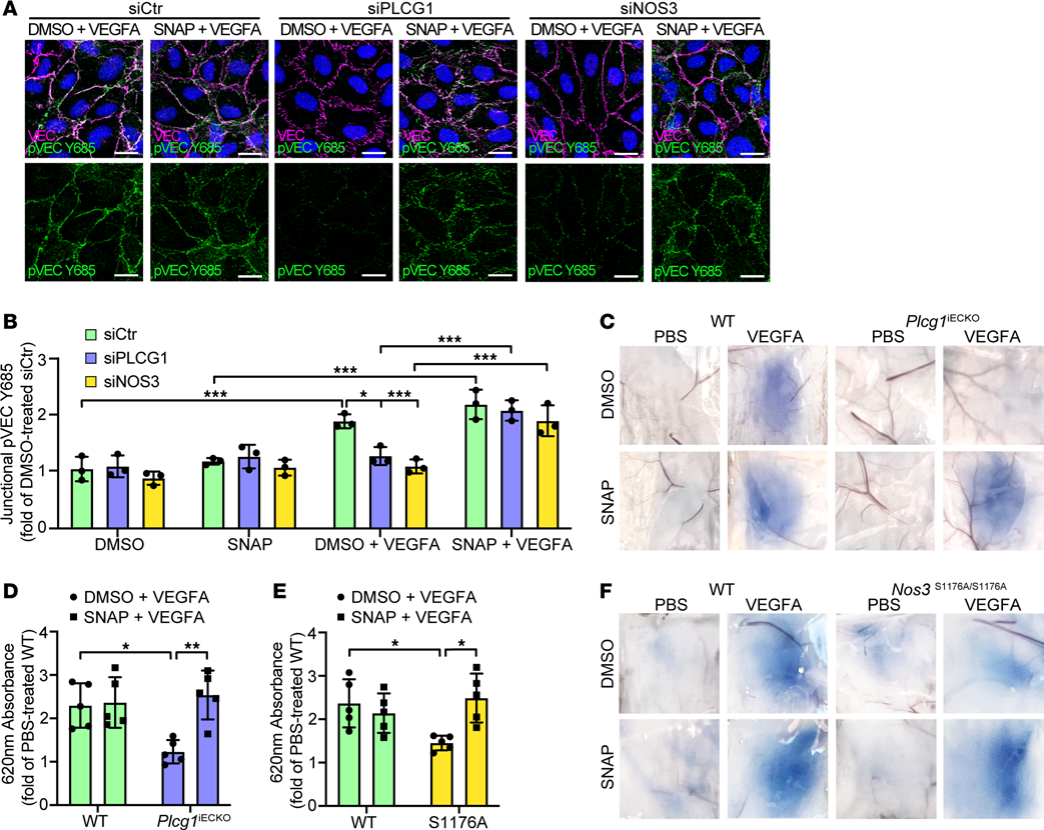
NO donor rescue of VE-cadherin phosphorylation and vascular leakage in the absence of PLCγ/eNOS signaling. (**A**) Representative images of immunostainings with antibodies against VE-cadherin (VEC) and pVEC Y685 of HUVECs treated with VEGFA + DMSO or VEGFA + SNAP for 5 minutes, pretreated with *siCtr*, *siPLCG1,* or *siNOS3*. Scale bar: 20 μm. (**B**) MFI quantification of data from **A** shown as fold of DMSO-treated *siCtr*. n= 3 independent experiments, ≥3 fields of view/experiment. 1-way ANOVA. (**C**) Miles assay showing Evans blue leakage in the back skin after intradermal injection of PBS or VEGFA, combined with DMSO (control) or the NO-donor SNAP, in tamoxifen-treated *Plcg1^fl/fl^; Cdh5-Cre^–^* (WT), and *Plcg1^fl/fl^; Cdh5-Cre^+^* (*Plcg1^iECKO^*) mice. (**D**) Quantification of extravasated Evans blue from **C** shown as fold change of PBS-treated WT mice, normalized to tissue weight; *n* = 5/genotype. 1-way ANOVA. (**E** and **F**) Quantification (**E**) and representative images (**F**) of Evans blue leakage in the back skin, in response to intradermal injection of PBS or VEGFA, cotreated with DMSO or SNAP, in *Nos3^+/+^* (WT) and *Nos3^S1176A/S1167A^* (S1176A) mice; *n* = 5/genotype. 1-way ANOVA. Data represent mean ± SD. *P < 0.05, **P < 0.01, ***P < 0.001.

The critical role of eNOS-mediated NO production in VEGF-induced vascular leakage was further validated by the in vitro and in vivo rescue effects of the NO donor S-nitroso-N-acetylpenicillamine (SNAP). SNAP-treatment of HUVECs silenced for either *PLCG1* or *NOS3* rescued VEGFA-induced VE-cadherin Y685 phosphorylation (Figure 8, A and B). Importantly, local administration of SNAP also rescued VEGFA-induced dermal vascular leakage in vivo (Figure 8, C–F) in the absence of either PLCγ or eNOS signaling.

## Discussion

Here, we have defined a VEGFR2 Y1173/PLCγ/eNOS/Src signaling pathway vital for VEGFA-induced macromolecular leakage in healthy vessels as well as in tumor vessels, of consequence for tumor immune status and disease outcome (see graphical abstract).

Mice heterozygous for the Y1173F mutation, mice lacking endothelial expression of PLCγ, or mice expressing a mutant eNOS S1176A, were all insensitive to VEGFA-induced vascular permeability. A prior study has indirectly associated PLCγ with vascular permeability, through DAG and PKC-induced phosphorylation of eNOS at S1176, required for eNOS activation (47). Subsequent generation of NO was described to enhance relaxation of vascular smooth muscle cells, leading to vessel dilation and increased blood flow and promoting extravasation of fluid and small molecules. However, eNOS/NO has also been described to enhance endothelial VE-cadherin phosphorylation (34, 48). Src has been found to interact directly with eNOS (49) and its activity has been placed both upstream (50) and downstream of eNOS, with NO-dependent modification of cysteine or tyrosine residues in c-Src, correlating with increased Src kinase activity (45, 46). We have previously shown that VEGFA-induced activation of SFKs is dependent on the adaptor molecule TSAd, which binds to the pY949 phosphosite in VEGFR2 through its SH2 domain and to Src’s SH2 and SH3 domains via phosphotyrosine residues and proline-rich stretches, respectively (11). In *Vegfr2^Y949F/Y949F^* mice, *Tsad^–/–^*, *Tsad^fl/fl^*; *Cdh5-CreERT2,* and *Src^fl/fl^;Cdh-CreERT2* mice, VEGFA-induced macromolecular leakage is suppressed, validating the important role for the VEGFR2 pY949-TSAd-Src pathway in VEGFA-induced macromolecular leakage (11, 12, 51, 52). This pathway may also regulate accumulation of Src at adherens junctions, where VEGFR2 exists in complex with VE-cadherin (12). Thus, these data suggest that Src activation at endothelial junctions requires both the VEGFA/VEGFR2 pY949 and pY1173 pathways, leading to VE-cadherin phosphorylation and dismantling of adherens junctions. It is possible that VEGFR2 pY949-TSAd activity initiates the release of Src from autoinhibition, and also brings Src in proximity to eNOS, allowing NO-dependent tyrosine nitration, which may be a prerequisite for full activation of Src. This notion is supported by the fact that NO donors alone did not induce dermal leakage, but rescued VEGFA responsiveness in mice lacking endothelial *Plcg1* or expressing a catalytically inactive eNOS (see Figure 8, C–F). Further studies are needed to identify which tyrosine residue(s) — and potentially also cysteine residues — in Src that are modified by PLCγ/eNOS–dependent nitrosylation/nitration and how this regulates Src activity.

A major downstream effect of PLCγ in development is the activation of PKCs in response to DAG accumulation and further PKC-mediated activation of the ERK 1/2 pathway (17). Although we do not formally rule out a contribution of ERK1/2 in regulation of vascular permeability in the mature vasculature, we note that vascular density and endothelial survival remained unaffected in the dermis of *Vegfr2^Y1173F/+^* and *Vegfr2^Y1173F/–^* mice. However, as shown by Karaman et al. (53), the reliance on VEGFR2 signalling in the adult vasculature is highly organ-specific and it is possible that VEGFR2-dependent PLCγ-PKC-ERK1/2 activation is critical for vessel maintenance in certain organs not examined here. We also do not rule out that other signal transducers, including the adaptor Shb, may have contributed to the Y1173F mouse phenotype. Shb binds to pY1173 and relays signals to FAK, thereby contributing to barrier stability (54, 55).

In mice expressing a mutant *Vegfr2* allele (*Vegfr2^Y1173F/+^*) reducing the level of endothelial PLCγ activation by half, the growth rate of melanoma and fibrosarcoma were unaffected, while vascular leakage was markedly reduced, accompanied by better efficiency of low-dose chemotherapy in restricting tumor growth. Moreover, the immune profile of tumors in *Vegfr2^Y1173F/+^* mice was polarized toward antitumor immunity as judged from the reduced infiltration of Foxp3^+^ Tregs, while CD8 cytotoxic T cell infiltration was unaffected compared with tumors in WT mice. The change in immunosuppressive cytokine expression in the *Vegfr2^Y1173F/+^* tumor microenvironment, which exhibits reduced production of *Tgfb1*, *Tgfb2*, *Il10, Il12a, and Ebi3*, supports the notion that suppressed PLCγ signaling in the endothelium stabilizes the vascular barrier, boosting antitumor immunity. In agreement, treatment with agonistic CD40 antibodies dampened tumor growth more efficiently in *Vegfr2^Y1173F/+^* mice compared with WT mice. Whether the diminished B cell population in *Vegfr2^Y1173F/+^* tumors represents immunosuppressive regulatory B cell needs further analysis.

The relevance of the PLCγ-correlated mouse tumor vessel phenotype described here for human cancer is implied by the strikingly specific expression of PLCγ in the tumor vasculature in certain cancer forms, in particular in ccRCC. ccRCC is characterized by an inactivating mutation in the tumor suppressor gene vHL, excessive HIF1α/HIF2α signaling, and cytokine production, including VEGFA (56). Accordingly, ccRCC is one of the most vascularized and immunogenic cancers, and the combined use of vascular normalization therapies and immune checkpoint inhibition has shown clinical benefit. However, not all patients respond, and some even progress on treatment (56–58). The clinical relevance of immune cell subsets and how their infiltration is regulated is incompletely understood, and, surprisingly, high immune cell infiltration in kidney cancer, including T cells and B cells, has been correlated to worsened survival (59–61). Further studies using fresh tumor samples from large cohorts of patients with RCC are clearly necessary to correlate PLCγ signaling in the ccRCC endothelium with vascular leakage and immune cell infiltration. However, the mutual relationship between tumor-mediated vascular permeability and an immunosuppressed tumor microenvironment is established for a number of human cancer forms (1, 6). Thus, increased hypoxia promotes the expansion of Tregs and drives T cell exhaustion, promotes polarization of tumor promoting macrophages (TAMs), and accumulation of myeloid-derived suppressor cells (6, 62, 63). In the KIRC cohort of 510 patients with ccRCC studied here, high PLCγ expression correlated with angiogenic activity programs, poor prognosis, and mRNA transcripts defining immunosuppressive Tregs and cytokines. In agreement, Braun et al. showed enrichment of terminally exhausted CD8^+^ T cells and protumoral TAMs in patients with ccRCC with advanced, metastatic disease (64). To steer tumor immunity by supporting influx of antitumor reactive immune cell populations, which, in turn, may further support barrier stability (65), is an emerging concept. Data presented here suggest that PLCγ acts as a surrogate marker for a hyperpermeable vasculature and an immune-suppressed microenvironment, which can be exploited further for personalized treatment of RCC.

## Methods

Additional methods are provided in the Supplemental Methods.

### Cell culture.

HUVECs and human dermal microvascular ECs (HDMECs) (PromoCell) were cultured in endothelial cell growth medium MV2 (PromoCell) with supplements. Primary cells were used at low passages only. MAEs expressing mouse VEGFR2 (FLK1) or VEGF-R2 Y1173F (FLK1 Y1173F) were generated by lentiviral transduction of pFUGIE-FLK1 and pFUGIE-FLK1 Y1173F plasmids, as described previously (66, 67). MAE cells were cultured in DMEM GlutaMAX (Gibco), supplemented with 10% FBS. Cells were maintained at 37°C with 5% CO_2_. Cells were seeded in 6-well plates at 3.5 × 10^5^ cells/well, 8-well glass slide chambers at 7.5 × 10^4^ cells/well, or 96-well plates at 2 × 10^4^ cells/well (Sarstedt). Stimulation was performed subsequent to 3 hours of starvation in MV2 without supplements. VEGFA165 at a final concentration of 100 ng/mL (Peprotech), TG at a final concentration of 1 μM (Thermo Fisher Scientific) and SNAP at a final concentration of 100 μM (Merck) were added to cells at 37°C for 5 minutes, unless otherwise indicated (see figures and figure legends). To terminate treatments, cells were placed on ice and washed in PBS (Gibco). For treatments before stimulation, cells were incubated at 37°C in 5% CO_2_ for 15 minutes with 2.5 μM or 5 μM U73122 (Merck), 5 μM MAPTAM (Merk), or 30 minutes with 20 μM U0126 (Merck), 2 μM Wortmannin (Merck), 10 μM Go6983 (Abcam), or DMSO as a control.

### RNAi.

HUVECs were transfected with 10 nM *siPLCG1* (Merk), *siSRC* (Merk), *siNOS3* (Thermo Fisher Scientific), or control *siRNA* (Merk) using Lipofectamine (Thermo Fisher Scientific) and Opti-MEM(Gibco). Experiments were performed 48–72 hours after siRNA transfection.

### Ca^2+^ measurements.

Ca^2+^ measurements was performed according to manufacturer’s instructions using the Fluo-4 Direct Ca^2+^ assay kit (Thermo Fisher Scientific). In brief, HUVECs were seeded in 96-well plates and Fluo-4 direct Ca^2+^ reagent containing 1X Fluo-4 direct Ca^2+^ assay buffer and 1 mM probenecid was added for 1 hour at 37ºC in 5% CO_2_. When using U73122 or MAPTAM, drugs were added after 30 minutes at a concentration of 2.5 μM and 5 μM, respectively, and incubation proceeded for another 30 minutes. Fluorescence was measured at 494 nm (excitation) and 516 nm (emission), following stimulation with 100 ng/mL VEGFA or 1μm TG.

### Antibodies used for immunoblotting.

Commercial antibodies used at 1:1,000 dilution, unless otherwise indicated, includes: mouse anti phospho-eNOS (S1177) (BD Biosciences, 612393; 1:500), mouse anti-eNOS (Abcam, Ab76198), mouse anti-GAPDH (Merk, MAB374; 1:2,000), rabbit anti-Grb2 (Santa Cruz, sc-255), rabbit anti-PI3Kp85 (Millipore, 06-195), and mouse anti–VE-cadherin (Santa Cruz, sc-9989; 1:500). Rabbit anti–phospho-Akt (4858), rabbit anti-Akt (9272), rabbit anti–phospho-FAK (Tyr576/577) (3281s; 1:500), rabbit anti-FAK (3285s), rabbit anti–Phospho-p44/42 MAPK (Erk1/2) (Thr202/Tyr204) (9101s), mouse anti–p44/42 MAPK (Erk1/2) (4696s), rabbit anti–phospho-PLCγ1 (Tyr783) (2821s), rabbit anti-PLCγ1 (2822s), rabbit anti-PLCγ2 (3872), rabbit anti–phospho-SFK (Tyr416) (2101s; 1:500), rabbit anti-Src (2123s), rabbit anti-phospho-VEGFR2 (Tyr951) (2471s; 1:500), rabbit anti–phospho-VEGFR2 (Tyr1175) (2478s; 1:500), rabbit anti–phospho-VEGFR2 (Tyr1212) (2477s; 1:500), and rabbit anti-VEGFR2 (2479s) were all purchased from Cell signaling technology. Secondary antibodies used were HRP-conjugated anti-rabbit (Cytiva, NA934; 1:10,000) and anti-mouse IgG (Cytiva, NA931; 1:10,000).

### Antibodies used for immunofluorescent staining.

Commercial antibodies used at a 1:100 dilution included: goat anti-CD31 (R&D systems, AF3628), mouse anti-CD34 (Agilent Technologies, IR63261-2), mouse anti–phospho-eNOS (S1177) (BD Biosciences, 612393), rabbit anti-eNOS (Ab5589, Abcam), rabbit anti-phospho-PLCγ1 (Tyr783) (Cell signaling technology, 2821s), rabbit anti-PLCγ1 (Cell signaling technology, 2822s), and goat anti–VE-cadherin (R&D systems, AF1002). pVEC Y685 antibody used for in vivo and in vitro experiments was prepared by immunizing rabbits with phospho-peptides of the corresponding region in mouse VE-cadherin (New England Peptide). The specificity of the VE-cadherin pY685 antibody was verified by immunostaining of tissue from *Cdh5^Y685F/Y685F^* mice (51). Secondary antibodies conjugated with Alexa Fluor dyes were obtained from Thermo Fisher Scientific and Jackson Immuno Research Laboratories and used at 1:400.

### In situ PLA.

A PLA was performed following directions for the Duolink PLA fluorescence kit. HUVECs were grown in 8-well glass slides at 7.5 × 10^4^ cells/well. After treatment and stimulation, 2% PFA was added for 15 minutes at room temperature(rt). After fixation, cells were permeabilized for 20 minutes in TBS, 0.1% Triton X-100 followed by Duolink blocking solution for 1 hour at 37°C. Primary antibodies, rabbit anti–phospho-SFK (Tyr416) (Cell Signaling technology, 2101s), mouse anti-Src (Millipore, Clone GD11), mouse anti-Yes (BD Biosciences, 610376), and mouse anti–3-Nitrotyrosine (Abcam, Ab61392) were diluted 1:100 in Duolink antibody diluent and added at 4ºC overnight. PLUS and MINUS PLA probes against mouse and rabbit primary antibodies were applied, and ligation, rolling circle amplification, and detection with fluorescent probes were performed. Slides were counterstained with goat anti–VE-cadherin antibody (R&D systems, AF1002; 1:100).

### Animals.

The generation of *Vegfr2^Y1173F^* mice has been described previously (15). *Vegfr2^Y1173F/+^* mice were back-crossed more than 10 generations to C57Bl/6J and further crossed with *Vegfr2^fl/fl^; Cdh5-CreERT2*. *Vegfr2^fl/fl^* mice were from Jackson Laboratory (018977), and *Cdh5(PAC)-CreERT2* mice were a gift from Ralf Adams (Department of Tissue Morphogenesis, Max Planck Institute for Molecular Biomedicine, Münster, Germany). The *Plcg1^fl/fl^* mice were a gift from Renren Wen (Versiti Blood Research Institute, Milwaukee, Wisconsin, USA), and the *Nos3^S1176A/S1176A^* were a gift from William Sessa (Vascular Biology and Therapeutics Program, Department of Pharmacology, Yale University School of Medicine, New Haven, Connecticut, USA). *Plcg1^fl/fl^* mice were crossed with *Cdh5-CreERT2* mice to generate the *Plcg1^fl/fl^; Cdh5-CreERT2* strain (*Plcg1^iECKO^)*.

Mice were maintained in heterozygous crossings in ventilated cages with 2–5 mice per cage. Each experiment was conducted with at least 3 animals per genotype representing individual biological repeats. Both males and females, typically 8 weeks old, were included. Sample size was chosen to ensure reproducibility and allow stringent statistical analysis. Tamoxifen (Merck) was injected i.p. at 80 mg/kg for 5 consecutive days to induce Cre recombinase–mediated gene recombination and mice were allowed to rest for 5 days before conducting experiments.

### Intravital vascular leakage assay.

Intravital imaging of the mouse ear with intradermal injection was performed as described previously (28). Briefly, subsequent to i.v. administration of 2,000 kDa FITC-dextran (Merck), mice, aged 8–12 weeks, were anesthetized by i.p. injection of Ketamine/Xylazine and the ear was secured to a solid support. Mice were maintained at a 37°C body temperature during the experiment for a maximum of 90 minutes. Time-lapse imaging was performed using single-photon microscopy (Zeiss LSM 710) and a high N.A. water-immersion lens (CF175 apochromat 25xW N.A1.1, Nikon). A volume of approximately 0.1 μl VEGFA165 (100 ng/μl, Peprotech) was injected intradermally using a submicrometer capillary needle together with a 10 kDa TRITC-dextran tracer (Thermo Fisher Scientific). Leakage sites were identified as defined sites of concentrated 2,000 kDa. To assess VE-cadherin junction structure during VEGFA-induced vascular leakage, freeze substitution was conducted immediately following the onset of leakage and before immunofluorescent staining. The onset of leakage was determined by intravital microscopy by the appearance of extravascular fluorescent dextran. Mice were promptly sacrificed by cervical dislocation and the ear tissue removed and snap frozen in isopentane cooled in a liquid nitrogen bath. Ear tissue was then submerged in methanol cooled to –80˚C and incubated at –80˚C for 24 hours. Tubes containing ear tissue and methanol were then placed in a metal block cooled to –80˚C and allowed to slowly warm to 4˚C, followed by processing for immunostaining.

### Subcutaneous mouse tumor models.

B16F10 melanoma and T241 fibrosarcoma tumors were established as previously described (12). Briefly, 5 × 10^5^ cells mixed 1:1 with Matrigel (Corning) (100 μl) were injected s.c. in the flank of WT and *Vegfr2*^Y1173F/+^ mice. Tumor size was measured with a caliper every second day from D7 and collected before reaching the humane endpoint. To assess tumor leakage, mice were injected systemically with FITC-2000 kDa lysine fixable dextran (Tdb labs) and TRITC-70 kDa (Tdb labs) 4 hours before tumor collection. Following anaesthesia using Ketamine/Xylazine, intracardiac perfusion was performed with DPBS. Tumors were divided in half for immunostaining or extraction in formamide. Extraction of the 70 kDa dextran was performed as described above. For microscopic evaluation of the fixable dextran, tumors were immersed in 1% PFA overnight followed by 30% sucrose overnight before proceeding with OCT embedding and freeze sectioning of 8 μm sections. For chemotherapy treatment of tumor-bearing mice, a subtherapeutic dose of TMZ, with no effect on WT mice, was used. From D4 to D8, after tumor inoculation, TMZ (2% DMSO) was administered i.p. every day at a concentration of 5 mg/kg. For immunotherapy, mice were treated i.v. on D4, D7, and D10 with 100μg anti-CD40 antibody (Clone FGK4.5, BE016-2, BioXcell) or IgG2a isotype control (clone 2A3, BE0089, BioXcell). Tumor size was measured with a caliper every second day from D6 until the humane endpoints were reached and all tumors were collected.

### Flow cytometry analysis.

B16F10 tumors were collected at D12, weighed, and placed in ice cold PBS. Dissociation was performed using the mouse tumor dissociation kit for soft tumors (Miltenyi Biotec). Immune cells were labeled with CD45 MicroBeads (Miltenyi Biotec) and a MACS magnetic separator was used for isolation (Miltenyi Biotec). Isolated CD45^+^ cells were incubated with live/dead fixable dye (Invitrogen; 1:1,000) and Fc block rat anti-mouse CD16/32 (BD biosciences, clone 2.4G2; 1:1,000) for 20 minutes at 4ºC. Following washes with MACS buffer (PBS supplemented with 0.5% BSA, 2 mM EDTA, 2 mM L-Glutamate, 1 mM sodium pyruvate, 0.45% D-glucose, and nonessential and essential amino acids), cells were stained in MACS buffer with brilliant staining buffer plus (1:10, 566385, BD biosciences) and primary antibodies against extracellular markers. For extracellular staining only, cells were fixed with 2% PFA and then washed. For intracellular staining, cells were fixed with FOXP3/transcription factor staining buffer set (Invitrogen) and stained with Foxp3 antibody according to the manufacturer’s protocol. All antibodies are listed in Supplemental Table 4. Cells were assessed on a CytoFLEX LX flow cytometer (Beckman Coulter) and obtained data were analyzed with FlowJo software, version 10.8.1 (FlowJo LLC, BD Biosciences) (see Supplemental Data File 2 for gating strategies).

### qPCR.

Total mRNA was extracted from B16F10 tumors using the Rneasy Fibrous Tissue Mini Kit (74704, Qiagen) and cDNA synthesis was performed using the iScript Adv cDNA Kit for RT-qPCR (Bio-Rad), followed by qPCR using the SsoAdvanced Universal SYBR Green Supermix (Bio-Rad) according to manufacturer’s protocol. Gene expression was detected with the following primers: mouse *Actb* (housekeeping gene) sense (CACTATTGCCAACGAGCGG) and antisense (TCCATACCCAAGAAGGAAGGC); mouse *Ebi3* sense (CACGGTGCCCTACATGCTAA) and antisense (AGGGTCCGGCTTGATGATTC); mouse *Il10* sense (CTGGACAACATCCTGCTAACCG) and antisense (GGGCATCACTTCTACCAGGTAA); mouse *Il12a* sense (ATCACAGGGGACCAAACCAG) and antisense (CCAAGGCACAGGGTCATCAT); mouse *Tgfb1* sense (GGTTTGTGGCTCCCGAGGGC) and antisense (CAGCCCTGCTCACCGTCGTG); mouse *Tgfb2* sense (CACACACACACACACACACACC) and antisense (TCCAAAGCTCGCCAACCATCAC); mouse *Tgfb3* sense (AAAGGATCACCACAACCCACAC) and antisense (TCTTCTTCCTCTGACTGCCCTG); mouse *Ifng* sense (CACGGCACAGTCATTGAAAG) and antisense (TTCCACATCTATGCCACTTGA); and mouse *Tnfa* sense (CTGTAGCCCACGTCGTAGC) and antisense (TTGAGATCCATGCCGTTG).

### Patient cohorts.

TMAs containing 5 different cancer forms, including kidney cancer, skin cancer, melanoma, lung carcinoma — both squamous and adenocarcinoma — and pancreatic ductal adenocarcinoma (PDAC) were generated by the Human Protein Atlas (HPA) consortium. The TMAs contained formalin-fixed, paraffin-embedded tumor tissue cores and were sectioned to 4 μm thickness and mounted on Superfrost Plus microscope slides (Thermo Fisher Scientific). Each cancer cohort consisted of 2 cores per patient from 12 patients.

*PLCG1* transcript level and patient survival correlations were performed using a cohort of patients with ccRCC with publicly available transcriptome data (KIRC), generated by the TCGA consortium (http://cancergenome.nih.gov/). The data set consists of 534 patients with ccRCC, with publicly available transcriptome data from 510 patients. For survival analysis, the data set was first subdivided using quartiles in 4 equally large patient groups, and the cutoff for low or high *PLCG1* expression was thereafter set to below and above the third quartile. Spearman correlations were used to explore the relationship between *PLCG1* transcript abundance and immune markers. Details about clinicopathological characteristics of the KIRC cohort is presented in Supplemental Table 1.

Bulk RNA-Seq STAR-counts data were downloaded from the TCGA database (https://portal.gdc.cancer.gov/) and used for expression analyses. The “DESeq2” package (v1.40.1) (68,) based on negative binomial distribution, was used to normalize raw count data and assess differential gene expression in *PLCG1*-high versus *PLCG1*-low groups, with age, sex, stage, and tissue source as covariates. Differentially expressed genes were filtered to give lists of genes with an adjusted *P* value less than 0.05 and with a log_2_ fold change of more than 1.0 or less than –1.0. Gene set enrichment analysis (GSEA) and visualization was performed using the clusterProfiler package (v4.8.1) (69, 70), using the Wald statistic as the ranking metric. R software (version 4.3.0) was used for all analyses.

### Statistics.

Statistical analysis was performed with GraphPad Prism 9 software or the statistical package SPSS 21.0. For comparison of means, significance was determined using unpaired 2-tailed Student’s *t* test, or a 1-way or 2-way ANOVA with Tukey’s multiple comparisons test. Data are expressed as mean ± SD and at least 3 independent biological repeats were performed for each experiment. For animal experiments, no statistical methods were used to predetermine sample size. The investigators were blinded to allocation during experiment and outcome assessment. χ^2^ test was used to determine associations of *PLCG1* expression and clinicopathological characteristics. Spearman rank correlation was used to explore correlation of gene expression. Log rank test and Kaplan-Meier analysis was used to estimate overall and disease specific survival in the KIRC data set. Cox proportional hazards model was used to compare HRs in both uni- and multivariable analyses. Data from multivariable analysis are shown as HR including 95% CI. *P* < 0.05 was considered statistically significant.

### Study approval.

Animal experiments were carried out in accordance guidelines from the Swedish Board of Agriculture and approved by the regional ethical committee, Uppsala, Sweden (permit 5.8.18-06789/2018). Tumor TMAs from HPA was covered by the HPA ethical permit (EPN Uppsala, Sweden, 2002/577, 2011/473). Written informed consent was received prior to study participation. The studies were performed in compliance with the 1975 Declaration of Helsinki, as revised in 1983.

### Data availability.

The data that support the findings of this study are available within the article and its Supplemental Tables and Figures. Values for all data points shown in graphs and values behind means are reported in the Supporting Data Values file. The MS proteomics data have been deposited to the ProteomeXchange Consortium via the PRIDE partner repository (71), and can be retrieved using the data set identifier PXD041024.

## Author contributions

ES and LCW conceptualized the project. ES, MM, MR, YD, CC, DC, EN, SP, DTL, and TN performed the experiments. ES, MM, AD, MR, EN, and LCW developed the methodology. DC, M. Shibuya, M. Simons, and LCW established the animal models. ES, MR, MM, M. Shibuya, M. Simons, AD, and LCW acquired funding for the project. ES and LCW drafted the mansucript. All authors edited the manuscript.

## Supplementary Material

Supplemental data

Supplemental data set 1

Supplemental data set 2

Supporting data values

## Figures and Tables

**Table 1:**
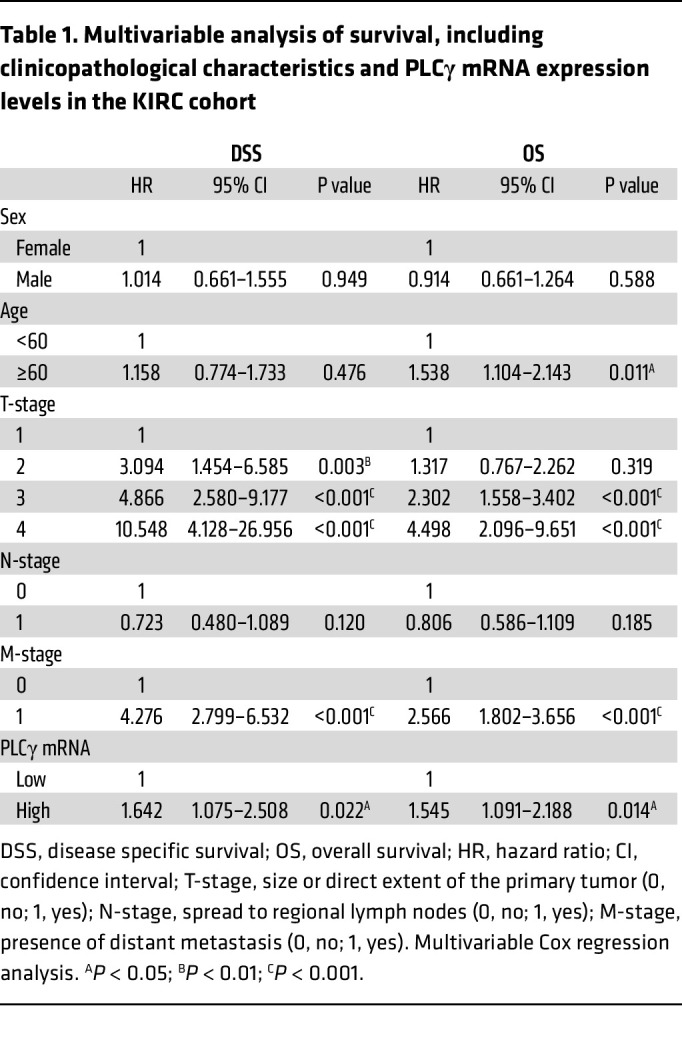
Multivariable analysis of survival, including clinicopathological characteristics and PLCγ mRNA expression levels in the KIRC cohort

## Version 1 08/31/2023

In-Press Preview

## Version 2 10/16/2023

Electronic publication
