# Supplementary material for: Endothelial VEGFR2-PLCγ signaling regulates vascular permeability and antitumor immunity through eNOS/Src
Source: J Clin Invest. 2023 Oct 16;133(20):e161366. doi: 10.1172/JCI161366 (PMC10575733; doi:10.1172/JCI161366)
Supplement: Supplemental data [file jci-133-161366-s019.pdf]

Supplemental Figures, Tables and Methods

Endothelial VEGFR2-PLCγ signaling regulates vascular permeability and anti-tumor immunity through eNOS/Src

Elin Sjöberg[1], Marit Melssen[1]*, Mark Richards[1]*, Yindi Ding[1], Catarina Chanoca[1], Dongying Chen[2], Emmanuel Nwadozi[1], Sagnik Pal[1], Dominic Love[1], Takeshi Ninchoji[1], Masabumi Shibuya[3], Michael Simons[2,4], Anna Dimberg[1], Lena Claesson-Welsh[1]

1. Department of Immunology, Genetics and Pathology, Beijer and Science for Life Laboratories, Uppsala University, Uppsala, Sweden
2. Yale Cardiovascular Research Center, Department of Internal Medicine, Yale University School of Medicine, New Haven, CT, USA
3. Institute of Physiology and Medicine, Jobu University, Takasaki, Gunma, Japan
4. Department of Cell Biology, Yale University School of Medicine, New Haven, CT, USA

<u>Content</u>

Supplemental Figures 1-7
Supplemental Tables 1-4
Supplemental Methods

Supplemental Figure 1

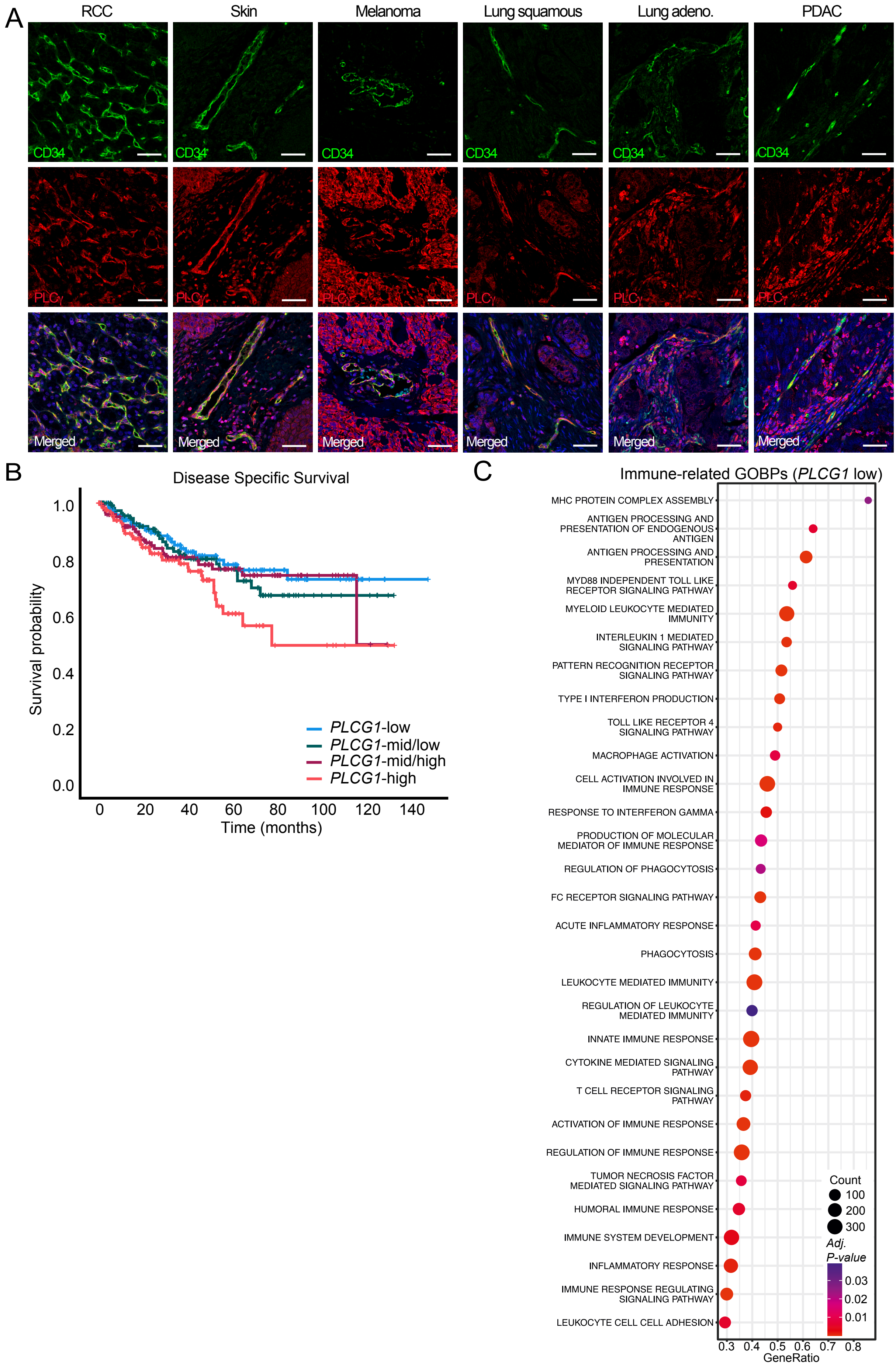

Supplemental Figure 1. Expression of PLCγ in human cancer.

(A) Representative images of CD34 (green) and PLCγ (red) immunostaining of formalin-fixed paraffin-embedded tumor biopsies from patients with renal cell carcinoma (RCC), skin cancer, melanoma, lung squamous carcinoma, lung adenocarcinoma and pancreatic ductal carcinoma (PDAC). Scale bar, 50 μm. (B) Kaplan-Meier analysis of *PLCG1* mRNA expression, divided into subgroups based on quartiles, and disease specific survival in the TCGA dataset for ccRCC (KIRC).
(C) Ranking of all immune activity-related gene ontology biological processes (GOBPs) in the *PLCG1*-low subgroup based on significance (adjusted *P*-value shown as heatmap) and ratio of affected genes within each GOBP.

Supplemental Figure 2

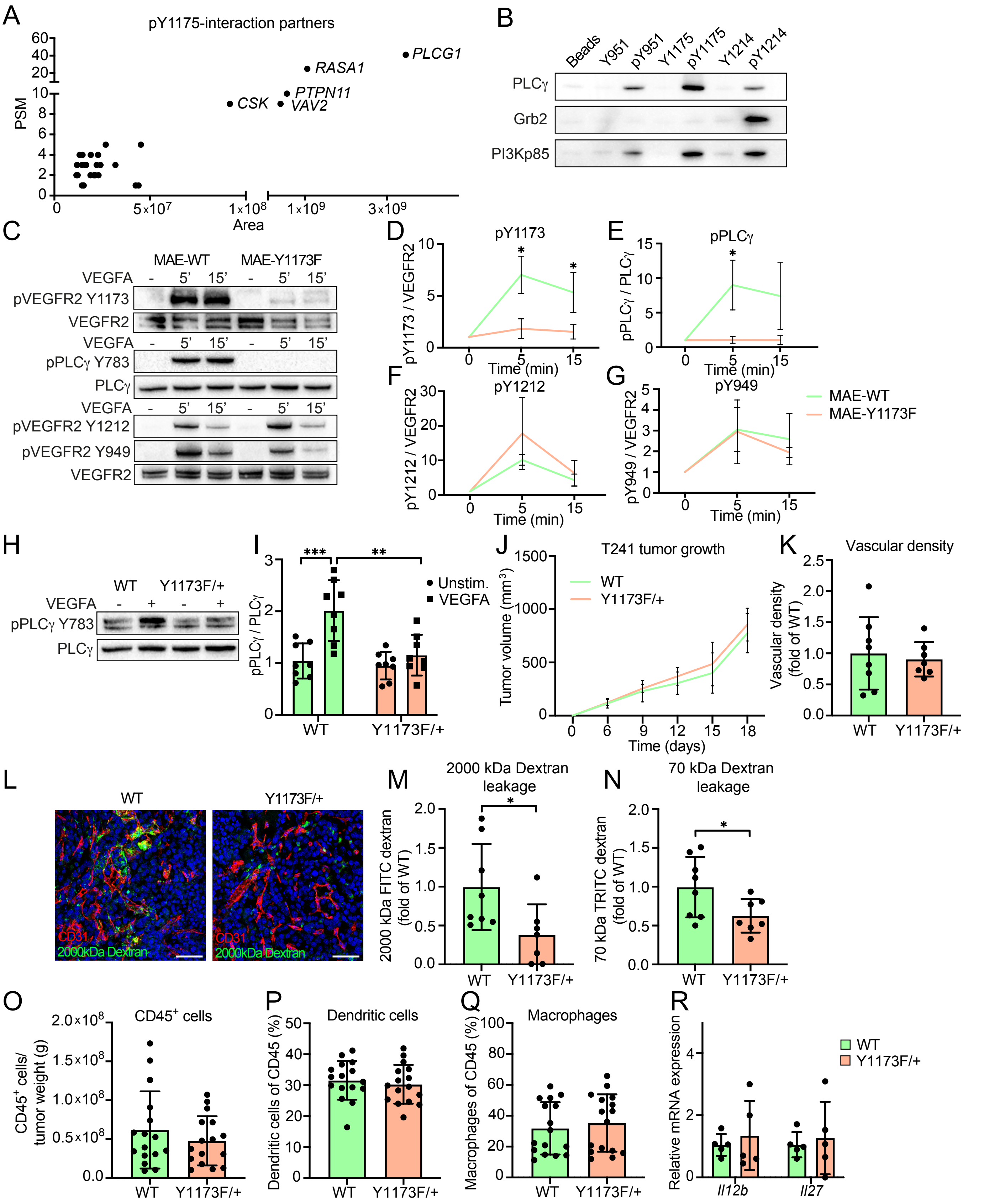

Supplemental Figure 2. PLCγ interacts with VEGFR2 pY1173/pY1175 to promote vascular leakage and immune cell infiltration in experimental tumors.

(A) Mass spectrometry detection of molecules captured by streptavidin pull-down of immobilized, biotinylated phospho-Y1175 peptides, pre-incubated with human dermal microvascular endothelial cell (HDMEC) lysates.
(B) Validation of findings in A, by pull-down of peptides (human Y951, Y1175, Y1214), followed by western blot (WB) analysis with antibodies against PLCγ, Grb2 and PI3Kp85.
(C) Representative WB to detect phosphorylation of VEGFR2 pY1173, pY1212, pY949 and PLCγ pY783 in mouse aortic endothelial (MAE)-WT and MAE-Y1173F cells treated with VEGFA (100 ng/ml) or not, for 5 or 15 min.
(D-G) Quantification of at least three independent experiments from C, for pY1173 (D), pPLCγ (E) pY1212 (F), pY949 (G), shown as fold of unstimulated. One-way ANOVA.
(H, I) Representative WB (H) and quantification (I) of pPLCγ Y783 in cultured lung endothelial cells isolated from *Vegfr2*$^{+/+}$ (WT) and *Vegfr2*$^{Y1173F/+}$ (Y1173F/+) mice, and treated with 100 ng/ml VEGFA or not; n=8 mice/genotype.
(J) Growth over time of T241 fibrosarcoma tumors in WT and Y1173F/+ mice; n=8 (WT) and 7 (Y1173F/+) mice. One-way ANOVA.
(K) Quantification of vessel density (mean fluorescent intensity) in WT and Y1173F/+ T241 tumors. ≥3 fields of view/tumor. One-way ANOVA.
(L) Representative images of leakage of a fixable 2000kDa FITC-labeled dextran (green) in T241 tumors. Vessels are labeled with CD31(red). Scale bar, 100 μm.
(M) Quantification of FITC-dextran in T241 tumors from L; n=8 (WT) and 7 (Y1173F/+) mice, ≥3 fields of view/experiment. One-way ANOVA.
(N) Fluorescent intensity measurement of extravasated 70kDa TRITC-conjugated dextran extracted from WT and Y1173F/+ T241 tumors; n=8 (WT) and 7 (Y1173F/+) mice. One-way ANOVA.
(O) Number of $CD45^+$ cells per gram tumor in B16F10 melanoma tumors of WT and Y1173F/+ mice harvested at day 12; n=16 mice/genotype. Unpaired two-tailed Students' t test.
(P, Q) Percentage of dendritic cells (CD11c and MHC class II co-staining) (P), and macrophages (CD11b and F4/80 co-staining) (Q), of the $CD45^+$ immune cell population in B16F10 tumors from WT and Y1173F/+ mice, harvested at day 12; n=16 mice/genotype. Unpaired two-tailed Students' t test.
(R) mRNA expression of *Il12b* and *Il27* in WT and Y1173F/+ B16F10 tumors; n=5 mice/ genotype. Unpaired two-tailed Students' t test.
Data represent mean ± SD. *P < 0.05, **P < 0.01, ***P < 0.001.

Supplemental Figure 3

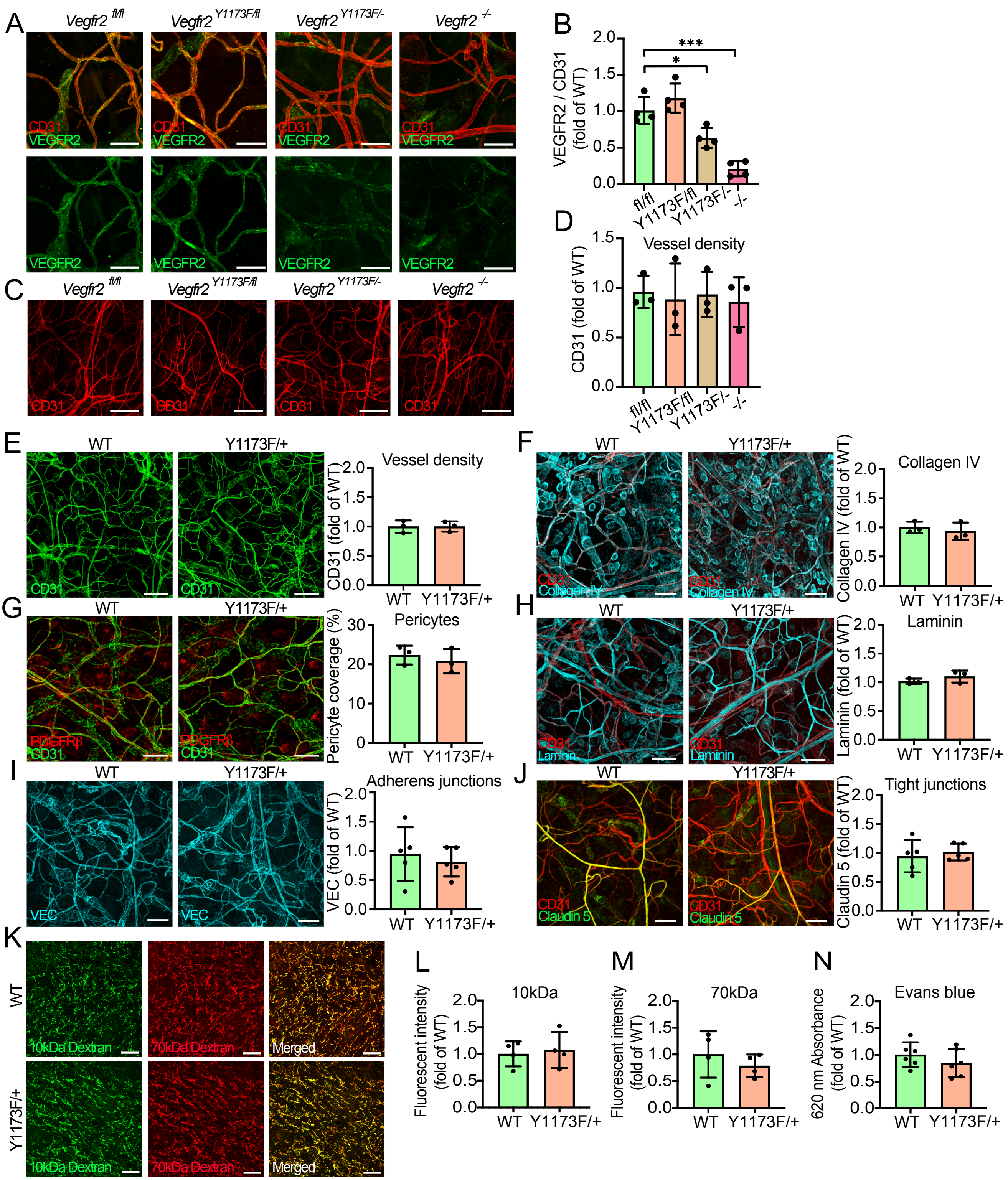

Supplemental Figure 3. *Vegfr2$^{Y1173F/+}$*mice display normal vascular markers and basal vascular permeability.

(A) Tamoxifen-induced *Vegfr2* removal confirmed by immunostaining for CD31 (red) and VEGFR2 (green) of ear dermis from *Vegfr2$^{fl/fl}$; Cdh5-Cre* - (fl/fl), *Vegfr2$^{Y1173F/fl}$; Cdh5-Cre* - (Y1173F/fl), *Vegfr2$^{Y1173F/fl}$; Cdh5-Cre* + (Y1173F/-) and *Vegfr2$^{fl/fl}$; Cdh5-Cre* + (-/-), mice. Scale bar, 50 μm.
(B) Quantification of VEGFR2 mean fluorescent intensity (MFI) from A; n=4 mice/genotype, ≥2 fields of view/experiment. One-way ANOVA.
(C, D) Representative images of vessel density (C) and MFI quantification (D) of long term *Vegfr2* excision (30 days) in the ear dermis of tamoxifen-treated *Vegfr2$^{fl/fl}$; Cdh5-Cre* - (fl/fl), *Vegfr2$^{Y1173F/fl}$; Cdh5-Cre* - (Y1173F/fl), *Vegfr2$^{Y1173F/fl}$; Cdh5-Cre* + (Y1173F/-) and *Vegfr2$^{fl/fl}$; Cdh5-Cre* + (-/-) mice; n=3 mice/genotype, ≥3 fields of view/experiment. One-way ANOVA. Scale bar, 200 μm.
(E-J) Immunostainings and quantifications of vessel density (CD31) (E). Scale bar, 200 μm, Collagen VI (F), pericyte coverage (PDGFRβ) (G), Laminin (H), Adherens junctions (VE-cadherin, VEC) (I), Tight junctions (Claudin 5) (J) in *Vegfr2$^{+/+}$* (WT) and *Vegfr2$^{Y1173F/+}$* (Y1173F/+) mice; n ≥3 mice/genotype, ≥3 fields of view/experiment. Scale bar, 100 μm. Unpaired two-tailed Students' t test.
(K-N) Representative images (K) and MFI quantifications of 10 kDa (L), 70 kDa (M) and Evans blue (N) basal dextran leakage in the ear of WT and Y1173F/+ mice; n ≥4 mice/genotype, ≥3 fields of view/experiment. Scale bar, 50 μm. Unpaired two-tailed Students' t test.
Data represent mean ± SD. *P < 0.05, ***P < 0.001.

Supplemental Figure 4

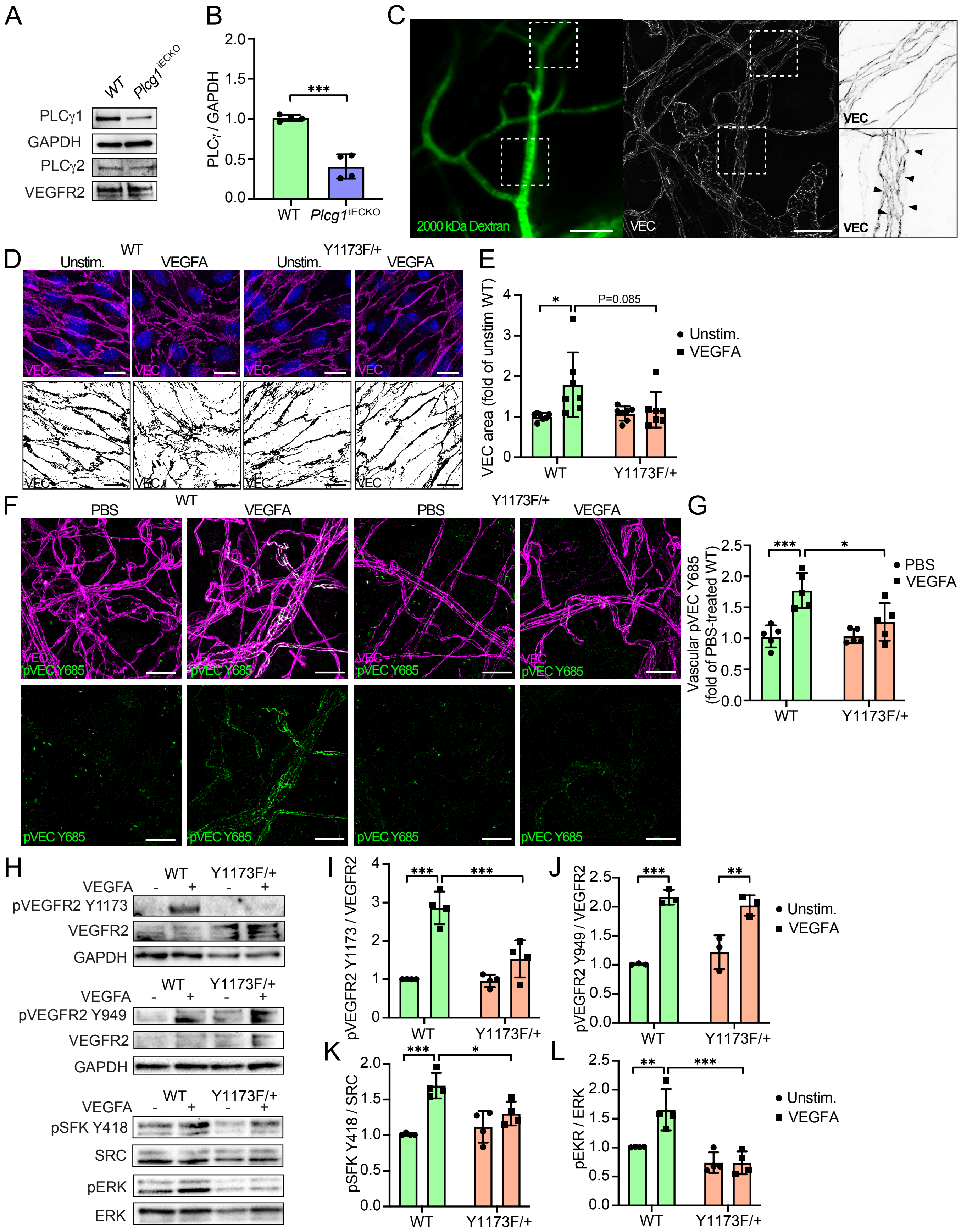

Supplemental Figure 4. Altered VEGFA-induced junctional disruption and SFK activation in *Vegfr2*$^{Y1173F/+}$ mice.

(A, B) Validation of endothelial PLCγ removal subsequent to tamoxifen administration in *Plcg1*^{iECKO} mice. Representative blot (A) and quantification (B), n=4 mice/genotype. Unpaired two-tailed Students' t test.
(C) Immunofluorescent staining for VEC (white; insets: black) in the ear dermis of snap frozen tissue subsequent to local VEGFA injection and analysis of 2000 kDa dextran leakage. Insets show VEC phenotype in vessels where leakage have occurred (arrowheads in lower panel) or not (upper panel). Scale bar 50 μm.
(D) Immunofluorescent staining for VE-cadherin (VEC) in unstimulated or VEGFA-stimulated (100 ng/ml) isolated lung endothelial cells (iECs) from *Vegfr2*$^{Y1173F/+}$ (WT) and *Vegfr2*$^{Y1173F/+}$ (Y1173F/+) mice. Upper panel shows merged images with nuclei (DAPI, blue), lower panel shows VE-cadherin only. Scale bar, 20 μm.
(E) Quantification of mean fluorescent intensity (MFI) for VE-cadherin area from D; n=7 mice/genotype, ≥3 fields of view/experiment. One-way ANOVA.
(F) Immunofluorescence staining with antibodies against VE-cadherin (VEC; magenta) and pVEC Y685 (green) in the back skin of 8-week-old WT and Y1173F/+ mice after intradermal injection of PBS or VEGFA. Scale bar, 20 μm.
(G) Quantification of MFI values for pVEC Y685 from F; n=5 mice/genotype, ≥2 fields of view/experiment. One-way ANOVA.
(H-L) Representative western blots (H) and quantification of WT and Y1173F/+ iEC with antibodies against pVEGFR2 Y1173 (I), pVEGFR2 Y949 (J), pSFK Y418 (K) and pERK 1/2 (L); n ≥3 independent experiments. One-way ANOVA.
Data represent mean ± SD. *P < 0.05, **P < 0.01, ***P < 0.001.

Supplemental Figure 5

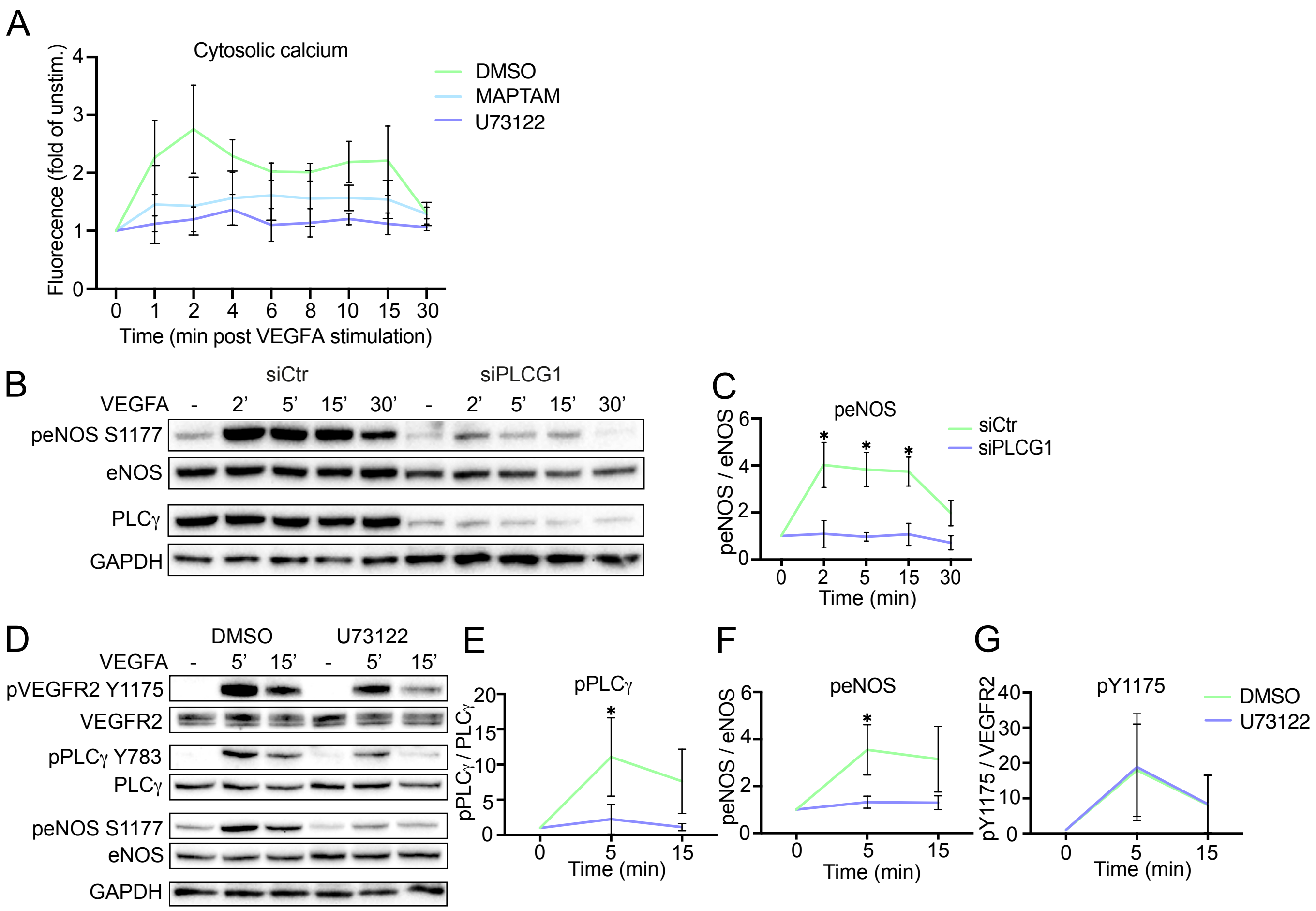

Supplemental Figure 5. PLCγ-mediated release of Ca^{2+} from ER enhances eNOS activity.

(A) Measurement of cytosolic Ca^{2+} levels using a Fluo-4 Direct™ Ca^{2+} assay in HUVECs pre-treated with DMSO (control), 5 μM MAPTAM or 2.5 μM U73122, followed by VEGFA stimulation for different time periods; n=5 independent experiments.
(B, C) Representative western blot (B) and quantification (C) of three independent experiments, showing phosphorylation and activation of eNOS in response to VEGFA stimulation over time, in HUVECs treated with siRNA against *PLCG1(siPLCG1)* or control siRNA (*siCtr)*. One-way ANOVA.
(D) Representative western blot showing VEGFA-induced signaling in HUVECs after treatment with DMSO (control) or 5 μM U73122.
(E-G) Quantification of at least three independent experiments to detect pPLCγ Y783 (E), peNOS S1177 (F) and pVEGFR2 Y1175 (G) from D, shown as fold of unstimulated. One-way ANOVA.
Data represent mean ± SD. *$P < 0.05$.

Supplemental Figure 6

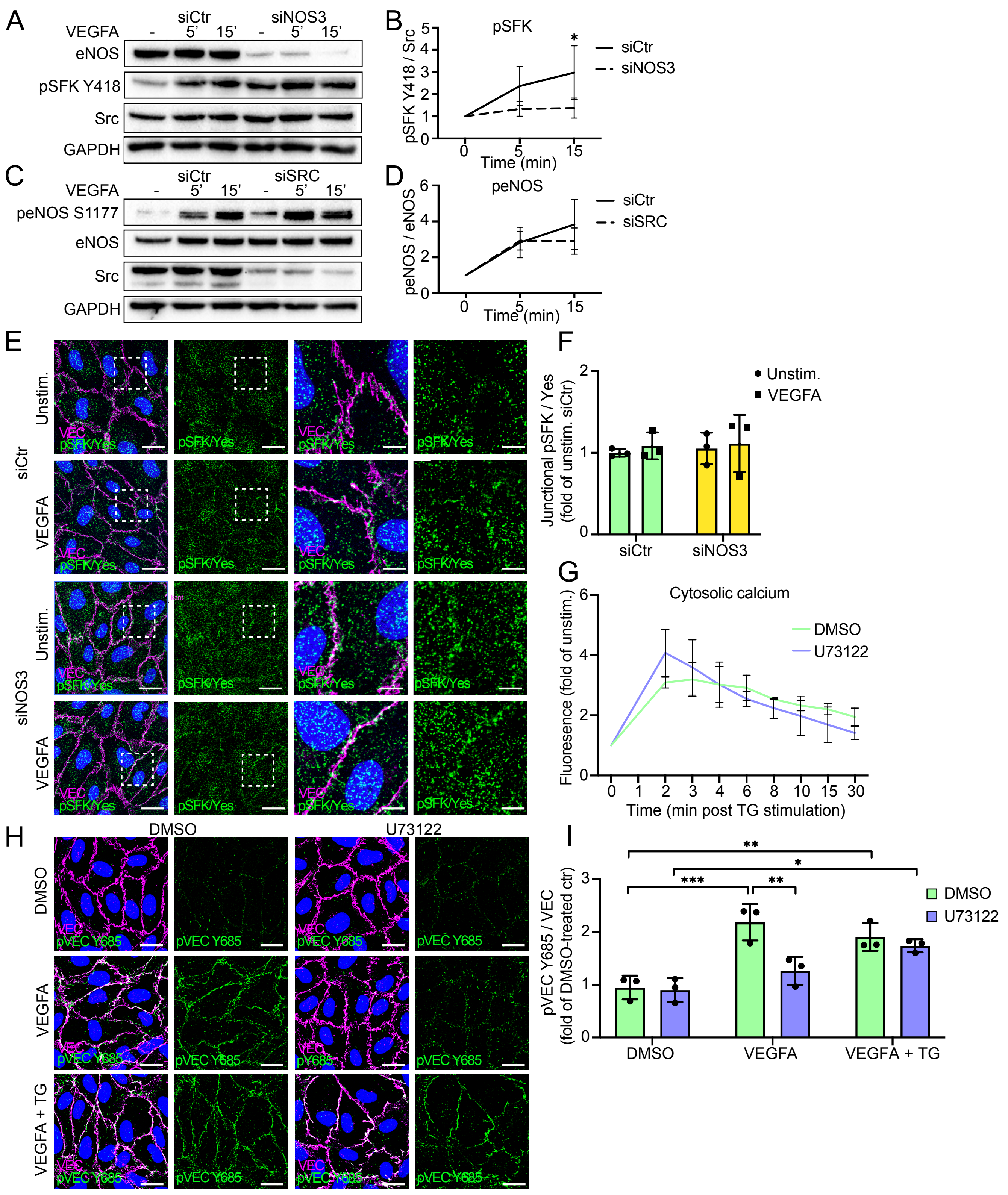

Supplemental Figure 6. VEGFA-induced PLCγ/eNOS/Src-signaling enhances VE-cadherin Y685 phosphorylation.

(A, B) Representative western blot (A) and quantification (B) of VEGFA-stimulated pSFK Y418 in *siCtr* and *siNOS3*-treated HUVECs; n=3 independent experiments. One-way ANOVA.
(C, D) Representative western blot (C) and quantification (D) of VEGFA-stimulated peNOS S1177 in *siCtr* and *siSRC*-treated HUVECs; n=3 independent experiments. One-way ANOVA.
(E) Proximity ligation assay (PLA) using antibodies against Yes and pSFK 418, visualizing phosphorylation of Yes (green), in HUVECs stimulated with VEGFA (100 ng/ml, 5 min), pre-treated with *siCtr* (two upper panels) or *siNOS3* (two lower panels). Junctions are stained for VE-cadherin (VEC; magenta) and nuclei with DAPI (blue). Scale bar, 20 μm. Boxed regions in left panels are magnified in panels to the right. Scale bar, 5 μm.
(F) Quantification of junctional PLA signals representing phosphorylated Yes from E, n=3 independent experiments, ≥3 fields of view/experiment. One-way ANOVA.
(G) Ca^{2+} measurements in HUVECs treated with 100 ng/ml VEGFA and 1 μM Thapsigargin (TG), pre-treated with DMSO (control) or 2.5 μM U73122; n=3 independent experiments.
(H) Immunofluorescent staining of VE-cadherin (VEC, magenta) and pVEC Y685 (green) in HUVECs treated with VEGFA, VEGFA + 1 μM TG or DMSO (control), subsequent to treatment with DMSO or 2.5 μM U73122. Nuclei stained with DAPI (blue). Scale bar, 20 μm.
(I) Quantification of junctional pVEC Y685 from H; n=3 independent experiments, ≥3 fields of view/experiment. One-way ANOVA.
Data represent mean ± SD. *$P < 0.05$, **$P < 0.01$, ***$P < 0.001$.

Supplemental Figure 7

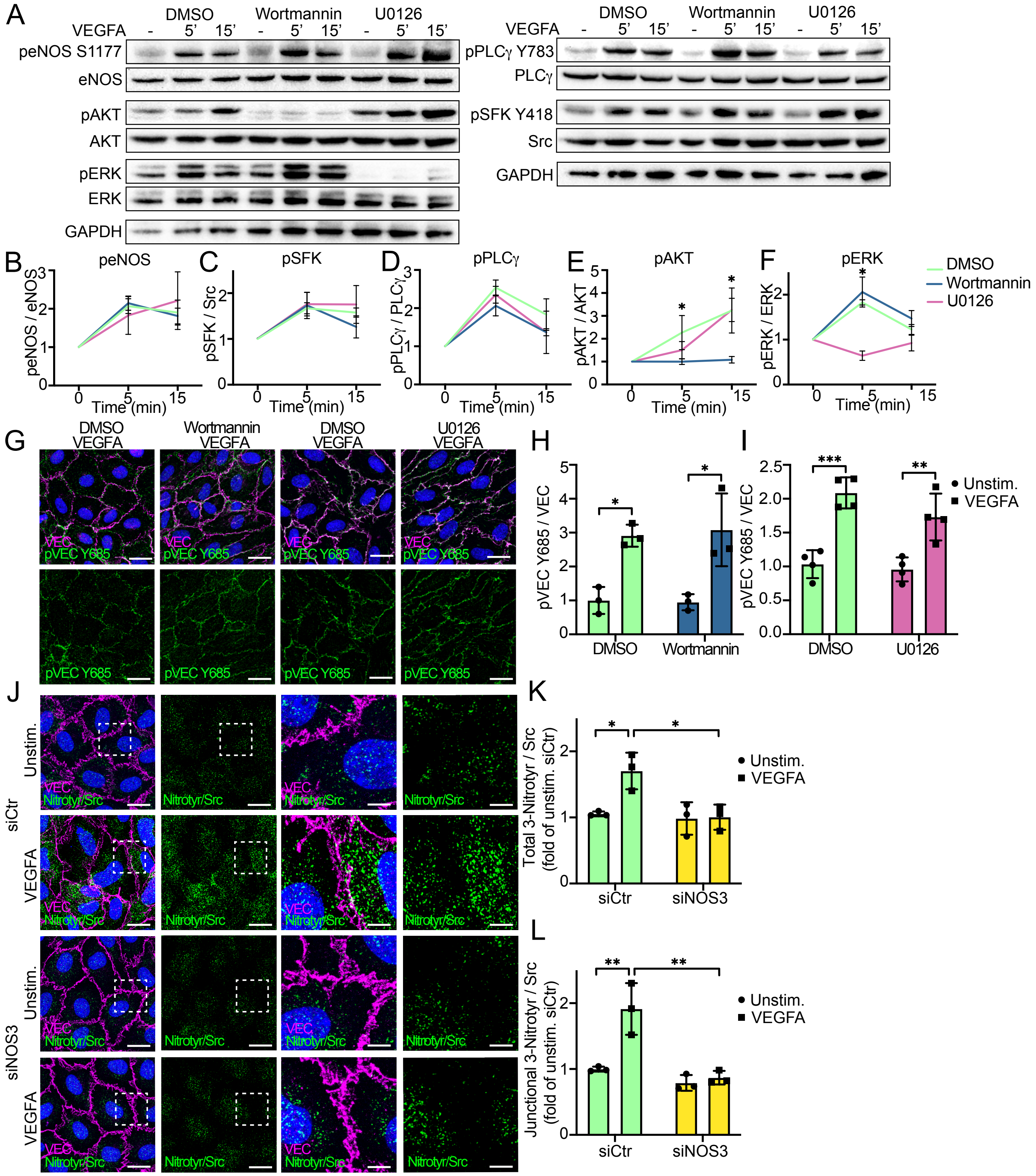

Supplemental Figure 7. eNOS stimulates tyrosine nitration of Src to induce vascular leakage.

(A) Representative western blot showing VEGFA-activated signaling in HUVECs pre-treated with DMSO (control), the PI3K inhibitor Wortmannin or the MEK inhibitor U0126.
(B-F) Quantification of at least three independent experiments from A, for peNOS S1177 (B), pSFK Y418 (C), pPLCγ Y783 (D), pAKT (E), pERK (F), shown as fold of unstimulated. One-way ANOVA.
(G) Immunostaining of VE-cadherin (VEC; magenta) and pVEC Y685 (green) in HUVECs, pre-treated with DMSO (control), Wortmannin or U0126 followed by stimulation with VEGFA (100 ng/ml, 5 min). Nuclei stained with DAPI (blue). Scale bar, 20 μm.
(H, I) Quantification of pVEC Y685 levels from G; n≥3 independent experiments, ≥3 fields of view/experiment. One-way ANOVA.
(J) Proximity ligation assay (PLA) using antibodies against 3-nitrotyrosine and Src, visualizing tyrosine nitration of Src (green) in HUVECs stimulated with VEGFA (100 ng/ml, 5 min), pre-treated with *siCtr* or *siNOS3*. Junctions stained for VE-cadherin (VEC; magenta) and nuclei with DAPI (blue). Scale bar, 20 μm. Boxed regions in left panels are magnified to the right. Scale bar, 5 μm.
(K, L) Quantifications of tyrosine nitration of total cellular Src (K) and of Src localized at adherens junctions (L) from J; n=3 independent experiments, ≥3 fields of view/experiment. One-way ANOVA.
Data represent mean ± SD. **P* < 0.05, ***P* < 0.01, ****P* < 0.001.

Supplemental Table 1. Associations of clinicopathological characteristics with PLCγ mRNA expression levels in the KIRC cohort.

	**Total =510**	***PLCG1*-low =383**	***PLCG1*-high =127**	***P*-value**
**Sex**				
Female	185	137	48	0.681
Male	325	246	79	
**Age**				
<60	237	172	65	0.219
≥60	273	211	62	
**T-stage**				
1	254	198	56	0.447
2	66	48	18	
3	179	130	49	
4	11	7	4	
**N-stage**				
0	228	172	56	0.873
1	282	211	71	
**M-stage**				
0	401	307	94	0.169
1	107	75	32	
Missing	2	1	1	

Abbreviations: T-stage = size or direct extent of the primary tumor. N-stage = spread to regional lymph nodes (0 = no, 1 = yes). M-stage = presence of distant metastasis (0 = no, 1 = yes). Statistical analysis: Pearson Chi square test.

**Supplemental Table 2.** Correlations of immune cell-markers with PLCγ mRNA expression in the KIRC cohort.		
	PLCG1	***P*-value**
CD19	r = 0.18	<0.001***
***MS4A1* (CD20)**	r = 0.21	<0.001***
CD4	r = 0.083	0.059
FOXP3	r = 0.141	0.001**
CD8A	r = 0.047	0.287
CD8B	r = 0.045	0.311
***ITGAX* (CD11c)**	r = 0.20	<0.001***
CD68	r = -0.16	<0.001***

Immune cell-markers; B-cells: CD19 and CD20, helper T-cells: CD4, regulatory T-cells: Foxp3, cytotoxic T-cells: CD8 dendritic cells: CD11c, macrophages: CD68. Statistical analysis: Spearman correlation. ** $P < 0.01$ *** $P < 0.001$

**Supplemental Table 3.** Correlations of inflammatory cytokines and PLCγ mRNA expression in the KIRC cohort.		
	PLCG1	***P*-value**
TGFB1	r = 0.242	<0.001***
TGFB2	r = 0.243	<0.001***
TGFB3	r = 0.454	<0.001***
IL12A	r = 0.308	<0.001***
EBI3	r = 0.042	0.343
IL10	r = -0.020	0.656
IFNG	r = 0.076	0.088
TNF	r = 0.096	0.030*
IL12B	r = 0.098	0.026*
IL27	r = 0.093	0.036*
Statistical analysis: Spearman correlation. * *P* <0.05 *** *P* <0.001		

**Supplemental Table 4.** Antibodies used for flow cytometry analysis.				
**Fluorochrome**	**Marker**	**Clone**	**Dilution**	**Company**
AF488	CD45	30-F11	1:200	Biolegend
PE	CD3	17A2	1:200	Biolegend
BV421	CD8	53-6.7	1:200	BD
BUV496	CD4	Rm4.5	1:500	BD
PE	CD25	3C7	1:200	BD
PE-Cy7	FOXP3	FJK-16s	1:100	Invitrogen
AF647	CD4	RM4-5	1:200	Biolegend
BUV395	CD3	145-2C11	1:100	BD
PE	CD11c	N418	1:500	Biolegend
BV421	MHC II	M5/114.15.2	1:500	Invitrogen
PE-Cy7	F4/80	Bm8	1:200	Biolegend
APC	CD19	6D5	1:200	Biolegend
BUV395	CD11b	M1/70	1:200	BD

Supplemental Methods

SDS-PAGE and Immunoblotting

Cells were lysed in RIPA buffer (ThermoFisher Scientific, 89901) supplemented with 50nM Na3VO4, Phosphatase inhibitor cocktail (Roche, 04906837001) and Protease inhibitor cocktail (Roche, 04693116001). LDS sample buffer (ThermoFisher Scientific, NP0007) and sample reducing agent (ThermoFisher Scientific, NP0009) were added and protein lysates incubated at 97ºC for 5 min prior to separation on NuPAGE 4-12% Bis-Tris gels (ThermoFisher Scientific) in MOPS SDS running buffer (ThermoFisher Scientific, NP0001), followed by transfer onto polyvinylidene difluoride (PVDF) membranes (ThermoFisher Scientific, 88518). Transfer was performed in NuPAGE transfer buffer (Novex, NP006). PageRuler Plus Prestained protein ladder (ThermoFisher Scientific, 26619) was used as a molecular weight marker. Membranes were blocked in 5% bovine serum albumin (BSA) (ThermoFisher Scientific) at room temperature (rt) for 1 h and incubated with primary antibodies overnight at 4ºC. Membranes were washed with Tris-buffered saline supplemented with 0.01% Tween 20 (TBS-T) (Merck) 3 x 10 min and incubated with secondary antibodies at rt for 1 h, and washed 3 x 10 min with TBS-T. Proteins were detected using ECL™ Prime Western blotting detection reagents (Cytiva, RPN2236) using a Bio-rad ChemiDoc MP. ImageJ/Fiji software was used for quantifications.

Mass spectrometry (MS) sample preparation

Synthetic peptides, phosphorylated or not, covering the Y951, Y1175 and Y1212 regions in human VEGFR2, were designed with an N-terminal biotin modification for pulldown using streptavidin-coated beads (1). Peptide sequences used for mass spectrometry (MS) analysis were hVEGFR2 Y1175: NAQQDGKDYIVLPISETLS and hVEGFR2 pY1175: NAQQDGKDpYIVLPISETLS. Sample preparation for MS was described earlier (1). Briefly, HDMEC cultures were lysed with hypotonic lysis buffer (10 mM HEPES pH 7.9, 2 mM $MgCl_2$, 10 mM KCl, 0.1% Na-deoxycholate) supplemented with Protease Inhibitor Cocktail (Roche) and 1 mM sodium orthovanadate. The cell suspension was passed through a syringe with narrow-gauge hypodermic needle (no. 27) five times with rapid strokes, and incubated under rotation at 4°C for 45 min. Mechanical lysis was repeated 10x followed by centrifugation at 11,000 *g* for 20 min. Two mg of cell lysate was incubated with the immobilized biotinylated peptides (1 nM) on streptavidin-coated Sera-Mag SpeedBeads (GE Healthcare) under rotation at 4°C for 1 h. The beads were collected using a magnet and washed with lysis buffer and dried. The dried beads were resuspended, trypsin digested and LC-MS runs was performed as previously described in detail (1). The MS analyses revealed retention of approximately 1500 proteins. Proteins detected in all conditions (bead-control, Y1175 and pY1175) were excluded before sorting for high peptide-spectrum match (PSM) >1 and area score. This selection identified proteins specifically enriched for binding to pY1175. For validation, pulldown was performed followed by elution in LDS sample buffer and WB analysis.

Immunofluorescent staining

For the human tumor tissue microarrays (TMAs), deparaffinization was performed 2 x 5 min stepwise in Xylene and EtOH (100%, 95%, 70%). Antigen epitopes were retrieved by microwave heating at 900 W, 5 min, followed by 500 W, 2 x 5 min in target retrieval buffer (pH 6, Dako).

Cells and mouse tumor sections were fixed in 1% paraformaldehyde (PFA), 15 min at rt and blocked with 1% BSA with 0.1% Triton X-100 for 1 h. Slides were incubated with primary antibodies at 4 ºC overnight. Following 3 x 15 min washes with 1X TBS with 0.1% Triton X-100, secondary antibodies were added for 1 h at rt. Slides were washed 3 x 15 min and mounted with DAPI Fluoromount-G® (SouthernBiotech, 0100-20). For whole mount stainings, tissues were fixed through intracardiac perfusion with 1% PFA followed by immersion in 1% PFA for 2 h at rt. Blocking was performed overnight at 4°C in TBS, 5% BSA, 0.2% Triton X-100. For immunostainings of whole mount of the back skin subsequent to intradermal injections, tissues were immediately fixed after the injection was performed.

Samples were incubated overnight with primary antibody, followed by washing in TBS, 0.2% Triton X-100 and incubation with appropriate secondary antibody for 2 h at rt before washing and mounting with fluorescent mounting medium (DAKO). For whole mount preparation of the back skin and ear skin, excess cartilage, fat and connective tissue were removed. Images were acquired using a Leica confocal microscope SP8 and HC PL CS2 20x, 40x and 63x objectives with 0.75 NA, 1.3 NA and 1.4 NA respectively, in a blinded fashion for animal experiments and unblinded for in vitro immunostainings. All images were analyzed using ImageJ/Fiji software.

The Miles assay and basal permeability

For the Miles assay, mice (8–10 weeks) were injected i.p. with pyrilamine maleate salt (4 mg/kg, Merck, P5514) diluted in 0.9% saline to inhibit histamine release, 30 min prior to tail vein injection of 1% Evans blue (100 µl in 0.9% saline). Evans blue was allowed to circulate for 10 min before intradermal (i.d.) injection in the back skin of recombinant mouse VEGFA165 (100 ng in 50 µl PBS), bradykinin (1 µg in 50 µl PBS) or histamine (250 ng in 50 µl PBS). To study the role of nitric oxide in VEGFA-induced permeability, mice were co-treated with VEGFA and the NO-donor SNAP (100ng in 50 µl PBS with 2% DMSO). The skin was excised 30 minutes subsequent to i.d injection. For histamine-induced permeability, mice were not pre-treated with pyrilamine maleate salt. For basal permeability assays, Evans blue, TRITC-conjugated 70 kDa (Tdb labs, TD70) and FITC-conjugated 10 kDa dextran (Tdb labs, FD10) (37.5 mg/kg body weight) were injected in the tail vein 3 h prior to organ collection. Before collection, mice were anaesthetised with Ketamine/Xylazine and intracardiac perfusion with DPBS was performed. Tissues were placed in formamide for extraction over night at 56°C and absorbance or fluorescence was measured using a spectrophotometer and normalized to tissue weight.

Isolation of lung endothelial cells

Mouse lungs were collected from postnatal day 10-12 and cells dissociated using the MACS dissociation kit (Miltenyi Biotec) according to manufacturer's instructions. In brief, lungs were collected on ice, rinsed and transferred to a gentleMACS C Tube containing the enzyme mix and dissociated on the gentleMACS dissociator. The endothelial cell population was labeled with CD31 MicroBeads (Miltenyi Biotec) and isolated on a MACS magnetic separator (Miltenyi Biotec). Endothelial cells captured on the beads were washed four times with PBS before elution and cells were suspended in MV2 growth medium including supplements and 5% penicillin/streptomycin (Merck). The isolated lung endothelial cells (iECs) were cultured on cover slips for immunostainings or in 6-well plates for immunoblotting. MV2 medium was changed every day for 5-7 days. When indicated, cells were treated with or without 100 ng/mL mouse VEGFA165 for 5 min and prepared as previously described for immunostainings or immunoblotting.

Reference

1. Testini C, Smith RO, Jin Y, Martinsson P, Sun Y, Hedlund M, et al. Myc-dependent endothelial proliferation is controlled by phosphotyrosine 1212 in VEGF receptor-2. *EMBO Rep.* 2019;20(11):e47845.
