# Supplementary figures and images for: Endothelial VEGFR2-PLCγ signaling regulates vascular permeability and antitumor immunity through eNOS/Src
Source: J Clin Invest. 2023 Oct 16;133(20):e161366. doi: 10.1172/JCI161366 (PMC10575733; doi:10.1172/JCI161366)

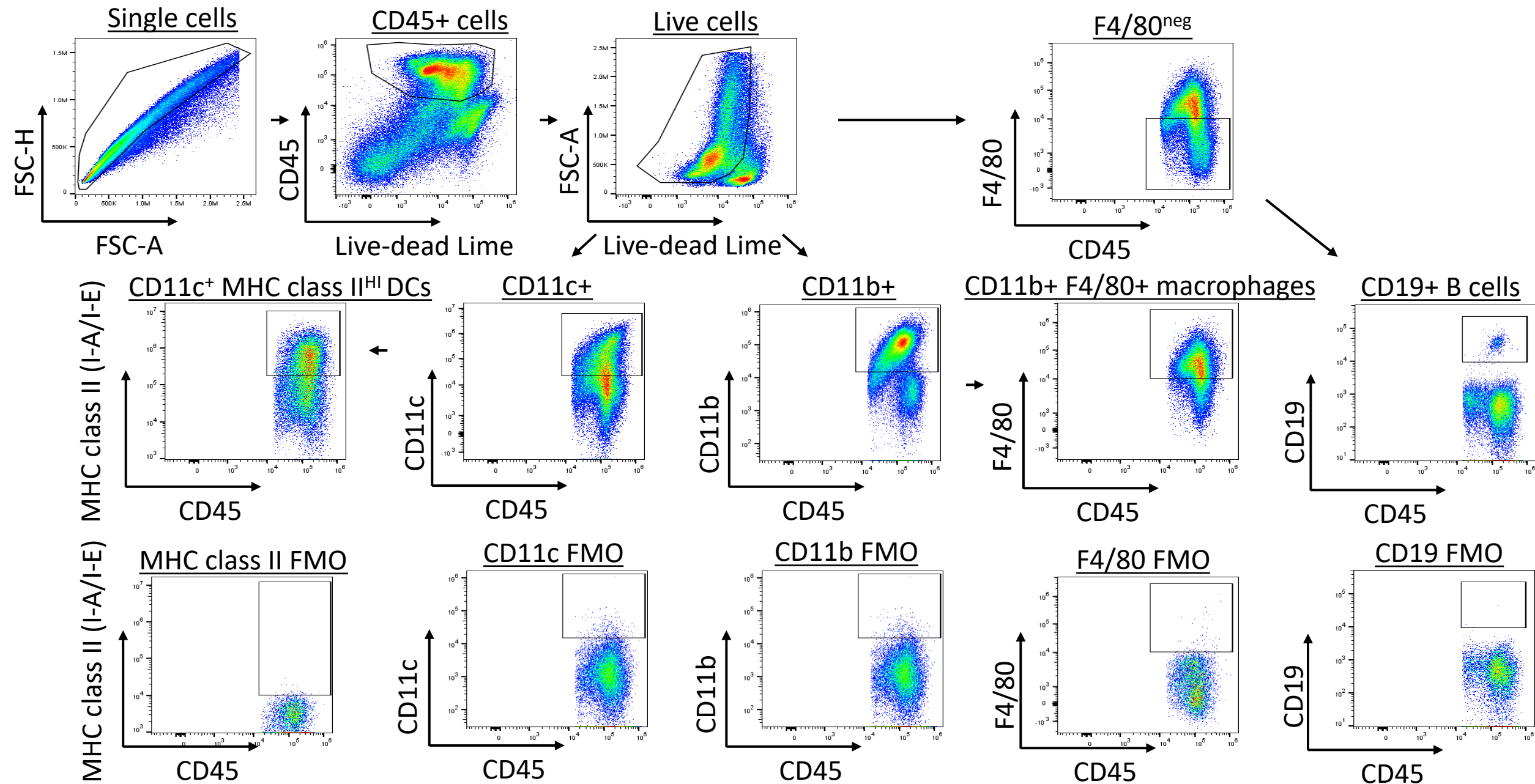

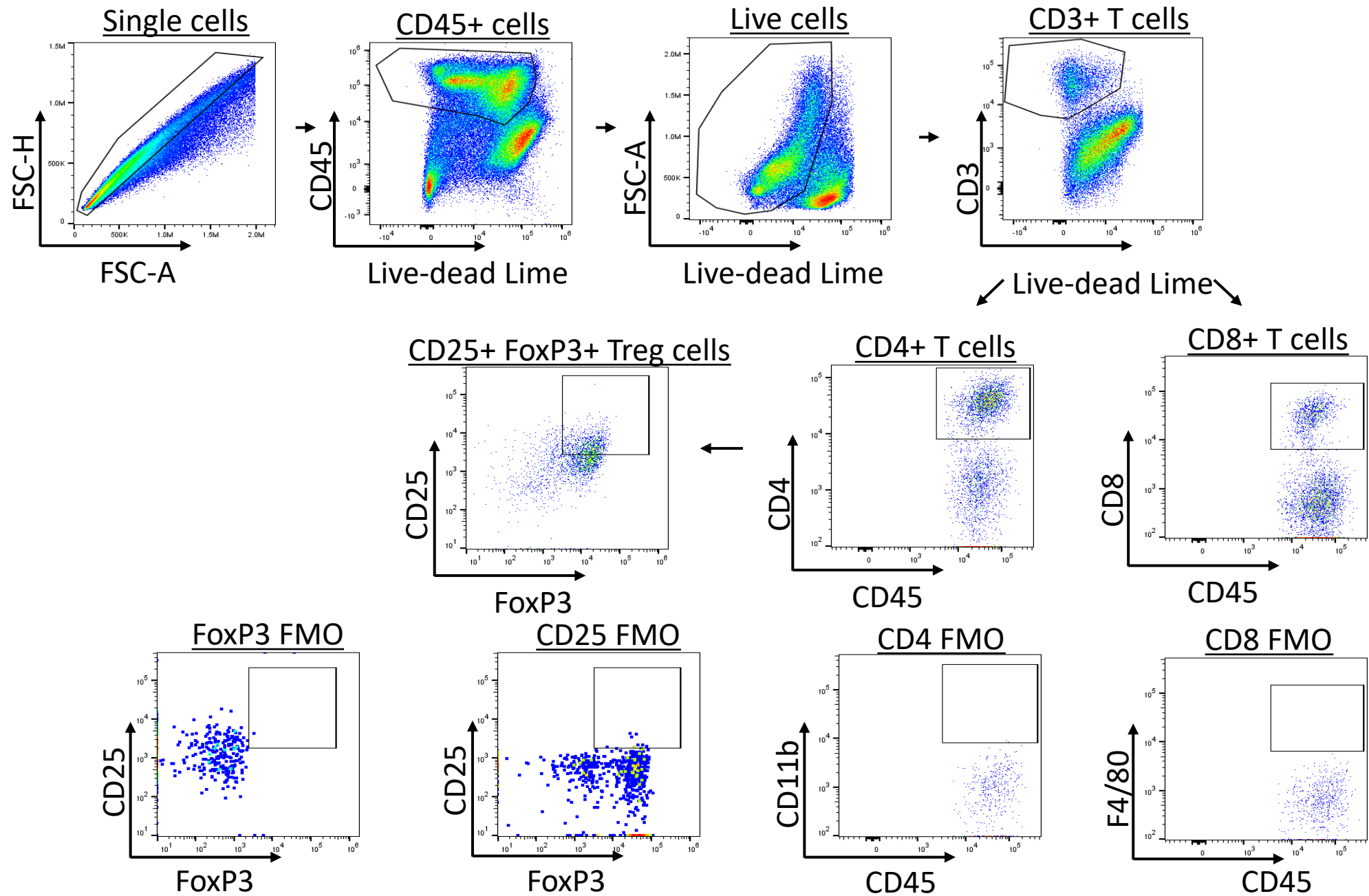

Supplement: Supplemental data set 2 [file jci-133-161366-s021.pdf]
